# A perturbation method for evaluating the magnetic field induced from an arbitrary, asymmetric ocean world analytically

**DOI:** 10.1016/j.icarus.2021.114840

**Published:** 2022-01-06

**Authors:** Marshall J. Styczinski, Steven D. Vance, Erika M. Harnett, Corey J. Cochrane

**Affiliations:** a Department of Physics, University of Washington, Box 351560, 3910 15th Ave NE, Seattle, WA 98195-1560, USA; b UW Astrobiology Program, University of Washington, Box 351580, 3910 15th Ave NE, Seattle, WA 98195-1580, USA; c Jet Propulsion Laboratory, California Institute of Technology, 4800 Oak Grove Dr, Pasadena, CA 91109-8001, USA; d Department of Earth and Space Sciences, University of Washington, Box 351310, 4000 15th Ave NE, Seattle, WA 98195-1310, USA

**Keywords:** Europa, Interiors, Magnetic fields, Satellites, Shapes

## Abstract

Magnetic investigations of icy moons have provided some of the most compelling evidence available confirming the presence of subsurface, liquid water oceans. In the exploration of ocean moons, especially Europa, there is a need for mathematical models capable of predicting the magnetic fields induced under a variety of conditions, including in the case of asymmetric oceans. Existing models are limited to either spherical symmetry or assume an ocean with infinite conductivity. In this work, we use a perturbation method to derive a semi-analytic result capable of determining the induced magnetic moments for an arbitrary layered body, provided each layer is nearly spherical. Crucially, we find that degree-2 tidal deformation results in changes to the induced dipole moments. We demonstrate application of our results to models of plausible asymmetry from the literature within the oceans of Europa and Miranda and the ionospheres of Callisto and Triton. For the models we consider, we find that in the asymmetric case, the induced magnetic field differs by more than 2 nT near the surface of Europa, 0.25–0.5 nT at 1 *R* above Miranda and Triton, and is essentially unchanged for Callisto. For Miranda and Triton, this difference is as much as 20%–30% of the induced field magnitude. If measurements near the moons can be made precisely to better than a few tenths of a nT, these values may be used by future spacecraft investigations to characterize asymmetry within the interior of icy moons.

## 1. Introduction

Any moon subjected to an oscillating magnetic field is a candidate for magnetic sounding—the determination of subsurface properties from magnetic measurements. Liquid water oceans containing dissolved salts conduct electricity, causing them to respond to oscillating fields that may be applied by their parent planet. This concept has proven especially useful in the study of icy moons in the outer solar system, as the induced magnetic fields that result from this interaction have been measured by orbiting spacecraft.

Induced magnetic fields observed by analysis of *Galileo* magnetometer data have provided the most compelling direct detection of oceans within Jupiter’s moons Europa, Ganymede, and Callisto (Kivelson et al., 2000; Zimmer et al., 2000; Kivelson et al., 2002). This demonstrated methodology will be deployed by the planned *Europa Clipper* and *JUICE* missions to characterize the oceans of these bodies. The same technique also shows promise for the investigation of the large moons of Uranus and Neptune (Khurana, 2019; Weiss et al., 2021; Cochrane et al., 2021; Arridge and Eggington, 2021); magnetometer investigations are likely to play a central role in future missions to these moons, too.

Magnetic sounding of moons requires a method for calculating the *expected* induced field that would result from a particular model of the interior conductivity structure, based on the measured oscillations of the parent planet’s magnetosphere far from the moon. Forward models of planetary magnetic induction typically assume a spherically symmetric body for simplicity, as in the oft-cited Parkinson (1983, Ch. 5). Various interior models can then be supposed, the results calculated, and induced fields compared against spacecraft measurements near the moon to determine which model best fits the measurements. However, to date there has not been any method available to analytically determine the induced magnetic field resulting from excitation of a planetary ocean that is not spherically symmetric. Analytic models are often faster to compute than numerical models, which is a considerable advantage for any large search of parameter space requiring many forward models.

The ability to suppose an asymmetric model for the conductivity structure of a moon is critical to an interpretation of measurements from upcoming missions, especially *Europa Clipper*, which plans flybys of Europa just 25 km above the surface—0.02*R*_*E*_ in altitude (Campagnola et al., 2019). Close to the moon, induced magnetic moments of quadrupole and higher order will have their largest effect relative to the dominant dipole moments. However, a spherically symmetric solution would predict only dipole moments are induced.

Further, asymmetries in the shape of subsurface oceans are fully expected, especially in the degree-2 shapes (*e.g. J*_2_ and *C*_22_ in the gravitational moments). The moons are expected to be triaxial ellipsoids because they spin as they orbit, and the asymmetric gravity field applied by their parent planet will introduce a bulging pattern. These shapes can even impact the induced dipole moments, by affecting where electric currents can flow within the body. Interpretation of magnetic measurements near icy moons will require an understanding of the effects asymmetry may have on the induced field. Sufficient measurements may even permit a detailed characterization of the asymmetric interior shape.

In this work, we refine our previously derived solution for highly conducting oceans (Styczinski and Harnett, 2021). Here, we generalize the approach to apply to cases of arbitrary conductivity, described by uniformly conducting layers with boundaries of arbitrary, but near-spherical shape^2^. Then, we apply our mathematical result to plausible models of asymmetry within the conductivity structure of the moons Europa, Miranda, Callisto, and Triton, informed by the literature and calculated using the geophysical modeling framework *PlanetProfile* (Vance et al., 2018, 2021). A summary of the results from the example cases studied is in Section 3.2; a full collection of the model results is contained in the Supplemental Text, Section S4.

The excitation field applied by the parent planet typically has complicated interactions with the asymmetric boundary shape, resulting in changes to the magnetic field that vary with latitude, longitude, and time. To simplify the analysis of the examples we study, we choose to evaluate the magnetic fields at the J2000 reference epoch, approximately noon UTC on January 1, 2000. “J2000” can refer to either the International Celestial Reference Frame (ICRF), with coordinate axes fixed at a point in time near noon UTC on January 1, 2000, or simply the time at which the ICRF axes are fixed. We use the instant of the J2000 epoch as an arbitrary point in time during the periods of oscillation in the excitation field applied to each of the studied moons, for the purpose of generating the figures in Section 3.2.

Plots showing the differences in all vector components of the induced field are included in the Supplemental Text (Figs. S1–S6). We include animations of the differences in induced magnetic field throughout the synodic period for some components as Supplemental Material. The Supplemental Material also contains Python code.^3^ for evaluating our model, including creation of all plots in this manuscript.

## 2. Methods

At core, we apply Maxwell’s laws at boundaries between regions and find the induced magnetic moments that satisfy the resulting equations. The same is true of the spherically symmetric, recursive solution developed by Srivastava (1966) and prominently applied by Zimmer et al. (2000), Seufert et al. (2011), and others. However, in our application, we describe the boundaries between regions of varying electrical conductivity by an arbitrary shape, rather than by a constant radius. The shape of the boundary has important consequences, and determines which magnetic moments are induced by a given excitation field applied by the parent planet. Here, we summarize the key points in our derivation and the final result for determining the induced magnetic field for an asymmetric conducting ocean.

A derivation of the Srivastava recursive method is recounted in more modern notation by the oft-cited Parkinson (1983). The Parkinson derivation has formed the basis for several influential studies of magnetic sounding of Europa, notably Zimmer et al. (2000), Khurana et al. (2002), and Hand and Chyba (2007). The Parkinson derivation contains several errors and inconsistencies that were repeated by the aforementioned studies, so we cover each step in our derivation from first principles and in fine detail in the Supplemental Text (Section S1). Despite the errors, the Parkinson derivation still gives the physically correct result for a spherically symmetric conductor. The consequences on the comparison to prior work that result from these errors are described in Section 2.1.1.

The key steps in our approach are:

1. Define a radial electrical conductivity structure within the body.
2. Apply Maxwell’s laws at the boundaries between uniformly conducting regions.
3. Expand the boundary radii in a first-order Taylor expansion in terms of spherical harmonics.
4. Solve the resulting linear system of equations for the induced magnetic moments.

Two main approximations are required for this approach to adequately describe a physical system.

First, we must assume that the electrical conductivity is a real-valued, uniform scalar within each layer within the planetary body. The imaginary part of the conductivity must be zero to obtain a steady-state solution for the inductive response, as we have assumed the oscillations are slow enough that displacement currents may be ignored. The assumption that the conductivity is uniform within each layer holds approximately for any planetary body with layers that are global in extent, although geophysical conditions changing with depth require a greater number of radial layers in order to be adequately represented (Vance et al., 2021). The consequence of this assumption is that our model is not expected to be capable of predicting the induced field of a body containing localized melt lenses (Schmidt et al., 2011), sills (Michaut and Manga, 2014), *etc.* within an ice shell.

Second, we assume that the boundaries are *near-spherical*, in that we may retain only the first term in a Taylor expansion about the boundary radius. This approximation is valid if the differences expected from the second-order terms are negligible, *i.e.* smaller than the expected uncertainty due to measurement precision. Second-order terms are expected to be negligible for all icy moons in the solar system. The validity of this approximation is discussed further in Section 2.4.1.

### 2.1. Basic physics of magnetic induction

In the presence of an electrical conductor, an oscillating magnetic field will induce a secondary, induced magnetic field in accordance with Maxwell’s laws. The strength of the induced field depends on the angular frequency *ω* of the oscillating applied field (called the *excitation* field) and the material properties everywhere within the conducting body: the electrical conductivity *σ* and the magnetic permeability *μ*. It is customary to assume *μ* = *μ_o_*, as this approximation holds very well on planetary scales (Saur et al., 2010). The faster the oscillation and the greater the electrical conductivity, the stronger the induced magnetic field, up to a maximum such that the radial component of the excitation field is exactly canceled at the outer boundary of a perfect conductor.

For simplicity in deriving our model, we neglect movement of conducting material within the body (as in the case of ocean currents) and rotation of the body within the external field, each of which can induce secondary fields (Saur et al., 2010; Vance et al., 2021). We also neglect currents outside the body. Although currents in the plasma environment around the body are not generally negligible, the principle of superposition permits us to consider each contribution to the net electromagnetic response independently—the net magnetic field is then the sum from each individual contribution. In this work, we consider only the induced magnetic moments generated by the interaction of the primary excitation field with the conducting body, as this is the dominant interaction that induces magnetic fields from within solar system moons. As a corollary, we must also assume that the induced fields are generally weak, such that they do not impact large-scale magnetospheric currents that partly determine the excitation field. A particularly strong induced magnetic field would create a feedback mechanism that would change the excitation field based on the properties of the inductive response, resulting in non-linear phenomena and solutions; we ignore such possibilities in this work.

Inside the conducting body, the net magnetic field **B** obeys a diffusion equation:

(1) $$\nabla^{2}B=\mu \sigma \frac{\partial B}{\partial t},$$

where *μ* is magnetic permeability and *σ* is electrical conductivity, both functions of position. For a sinusoidal time dependence $e^{-i\omega t}$ with angular frequency ω,

(2) $$\nabla^{2}B=-i\omega \mu \sigma B,$$

or alternatively

(3) $$\nabla^{2}B=-k^{2}B,$$

where *k* is known as the wavenumber. Eq. (3) is a vector Helmholtz equation, with

(4) $$k=\sqrt{i\omega \mu \sigma }.$$

Outside the body, both the excitation field and the induced field, as well as their sum, obey the Laplace equation:

(5) $$\nabla^{2}B=0.$$

In this outer region, the magnetic field can be determined from (Jackson, 1999)

(6) $$B_{\text{net}}=-\nabla \left[\sum_{n,m}R\left(B_{nm}^{e}{\left(\frac{r}{R}\right)}^{n}+B_{nm}^{i}{\left(\frac{R}{r}\right)}^{n+1}\right)Y_{nm}\left(\theta ,\phi \right)\right],$$

where the excitation moments $B_{nm}^{e}$ are determined by measuring the oscillating field far from the body, $B_{nm}^{i}$ are the induced magnetic moments, *R* is a unit of radial distance (typically the outer radius of the conducting body), and $Y_{nm}$ are the spherical harmonics.

As the induced field can only be as strong as the excitation field, and that only happens in the limit of high conductivity, it is often convenient to consider the ratio of the induced and excitation moments, $B_{nm}^{i}/B_{nm}^{e}$, sometimes called the “*Q*-response”. In the case of spherical symmetry and in the high-conductivity limit, this ratio approaches n/(n+1). Because $B_{nm}^{i}/B_{nm}^{e}$ are in general always complex, we define the complex amplitude $A_{n}^{e}$ such that

(7) $$B_{nm}^{i}=\frac{n}{n+1}A_{n}^{e}B_{nm}^{e}.$$

#### 2.1.1. Comparison to derivations used in prior work

In the commonly studied case of a uniform excitation field, with *n* = 1, $A_{n}^{e}$ may be expressed in terms of the real amplitude *A* and phase delay ϕ (*e.g.* as defined by Zimmer et al., 2000):

(8) $$A_{1}^{e}=Ae^{-i\phi },$$

allowing for ready comparison with prior work.

Note that the negative exponent in Eq. (8) results from our definition of *k* in Eq. (4), with +*i* under the square root, and the contrasting choice of Parkinson (1983)—and several authors of prior work who have relied on the Parkinson derivation, notably Zimmer et al. (2000), Khurana et al. (2002), Hand and Chyba (2007), and Arridge and Eggington (2021). The Parkinson (1983) derivation contains an error, in that choosing −*i* in the definition of *k* results in the *modified* spherical Bessel equation, with the *modified* spherical Bessel functions $i_{n}\left(kr\right)$ and $k_{n}\left(kr\right)$ as solutions, as detailed in Schilling et al. (2007) and Seufert et al. (2011). Parkinson (1983) incorrectly arrives at the standard spherical Bessel equation (Eq. S18). In effect, both our input wavenumber *k* and our result for the complex response amplitude *A* are equal to the complex conjugate of the analogous quantities from prior work, hence the negative exponent in Eq. (8). See Section S1.1 for more information. We define the relationship between $A_{1}^{e}$, *A*, and ϕ as in Eq. (8) to facilitate comparison with the rich set of prior work related to this topic.

While we note that $A_{1}^{e}$ may be compared directly to the real quantities used in prior work, we also wish to point out that for interpretations based on multilayer models, $A_{1}^{e}$ contains more detailed information. Past analyses (*e.g.* Khurana et al., 2002; Hand and Chyba, 2007) have focused on the information contained only in the amplitude while treating the ocean as a single, uniform conducting layer. As elaborated by Vance et al. (2021) and Cochrane et al. (2021), the amplitude and phase information contained in $A_{1}^{e}$ cause this quantity to occupy specific regions of the complex plane for different interior structure models. This structure-dependent behavior restricts degeneracies between models to single points, in contrast to the lines traced by degenerate response amplitudes *A* (*e.g.* Khurana et al., 2002, Fig. 5). Multilayer models can also incorporate additional constraints from geophysics and spacecraft measurements, as in the case of *PlanetProfile*. A detailed analysis of the complex response amplitude for different multilayer models is therefore capable of constraining plausible best-fit interior models, even considering only a single excitation period.

### 2.2. Radial conductivity structure

Within the planetary body, we must suppose a radial conductivity structure *σ*(*r*). Solution of the vector Helmholtz equation (Eq. (3)) in spherical coordinates is made a tractable problem by our assumption that each layer has uniform conductivity. In this work, we use the *PlanetProfile* framework (Vance et al., 2018, 2021) to model the electrical conductivity of ocean waters under geophysical conditions likely found within icy moons. The conductivity within ice and rock are typically small (< 10^−2^ S/m, Glover, 2015). The conductivity of an iron core may be much higher (> 10^6^ S/m, Khurana et al., 2002), but its contribution to the induction response is likely a few percent at most (Seufert et al., 2011) due to its smaller size and screening of the excitation field by the ocean. For the example cases we present in Section 3, we neglect the conductivity of ice, rock, and a possible iron core, but these are easily inserted into our model.

We require a spherically symmetric conductivity model as a starting point for our derivation because we assume only the boundaries between the conducting regions are asymmetric. This is a requirement for an analytic solution of the boundary condition equations. Although the conductivity within each asymmetric layer is likely to differ with a change in depth, our near-spherical approximation ensures that this difference is small compared to the global effect of the asymmetric boundary. This approximation is most valid when there are sharp contrasts in conductivity between the layers, *e.g.* between the ocean and nonconducting ice shell, because the depth-dependence of the conductivity in the ocean has a much smaller effect on conductivity than the layer transition.

### 2.3. Applying Maxwell’s laws at the boundaries

In spherical coordinates, the vector components of general solutions to Eqs. (1) and (5) (the diffusion and Laplace equations, respectively) are spherical harmonics and their *θ* derivatives, multiplied by either spherical Bessel functions and their *r* derivatives (inside) or a power series in *r* (outside) (Moffatt, 1978; Jackson, 1999). As we have assumed *μ* = *μ*_*o*_ everywhere, and currents are not confined to the boundary surfaces, Maxwell’s laws dictate that the vector components of **B** must be continuous at every boundary. This continuity gives us equalities that relate coefficients for the general solutions that describe each region, and ultimately will yield a solvable set of linear equations.

In this work, we use the fully normalized, complex spherical harmonics:

(9) $$Y_{nm}=\sqrt{\frac{2n+1}{4\pi }\frac{\left(n-m\right)!}{\left(n+m\right)!}}P_{n}^{m}\left(\cos\theta \right)e^{im\phi },$$

(10) $$Y_{n,-m}={\left(-1\right)}^{m}Y_{nm}^{*}$$

for degree *n* and order *m*, where $P_{n}^{m}$ are the associated Legendre functions with the Condon–Shortley phase (Condon and Shortley, 1951). In contrast, much of the geomagnetism community uses real-valued spherical harmonics, in terms of sinϕ and cosϕ, in the Schmidt normalization, and omit the Condon–Shortley phase. We have carefully selected a normalization for optimal clarity in the derivation. Expressing each harmonic as succinctly as possible is vital, including their derivatives, products with other harmonics, and linear combinations of harmonics. Each of these is made more complicated in the Schmidt normalization, so we use fully normalized harmonics to detail the mathematics. In the software we provide as supplemental material, we include an option to print the induced magnetic moments in the Schmidt normalization.

For the spherical harmonics, we define

(11) $$Y_{nm}^{⋆}\equiv \frac{\partial Y_{nm}}{\partial \theta }=\frac{1}{\sin\theta }\left(-w_{nm}^{-}Y_{n-1,m}+w_{nm}^{+}Y_{n+1,m}\right)\;\text{for}\;m\geq 0,$$

(12) $$w_{nm}^{-}=\left(n+1\right)\sqrt{\frac{n^{2}-m^{2}}{\left(2n-1\right)\left(2n+1\right)}},\;w_{nm}^{+}=n\sqrt{\frac{{\left(n+1\right)}^{2}-m^{2}}{\left(2n+1\right)\left(2n+3\right)}},$$

(13) $$Y_{n,-m}^{⋆}={\left(-1\right)}^{m}Y_{nm}^{⋆*},$$

where * denotes a complex conjugate. For spherical Bessel functions of the first and second kind $j_{n}\left(kr\right)$ and $y_{n}\left(kr\right)$, we define

(14) $$j_{n}^{⋆}\equiv \frac{d}{dr}\left(rj_{n}\right)=\left(n+1\right)j_{n}-kr\;j_{n+1},\;y_{n}^{⋆}\equiv \frac{d}{dr}\left(ry_{n}\right)=\left(n+1\right)y_{n}-kr\;y_{n+1}.$$

The starred functions appear in the tangential boundary condition equations and the overall solutions.

### 2.4. Near-spherical boundary shapes

We must define surfaces $r_{l}\left(\theta ,\phi \right)$ for arbitrary boundaries that we will insert into the internal and external boundary condition equations. We choose boundary surfaces of the form

(15) $$r_{l}\left(\theta ,\phi \right)={\bar{r}}_{l}+\varepsilon_{l}\sum_{p,q}\chi_{pq}^{l}Y_{pq}\left(\theta ,\phi \right)$$

because expanding in spherical harmonics will allow us to make use of well-known relations from the mathematics of quantum mechanics. Here, ${\bar{r}}_{l}$ is the mean radius of boundary *l*, *ε*_*l*_ is the amplitude of deviation from spherical symmetry, $\chi_{pq}^{l}$ is a dimensionless constant that indicates the relative amount of each harmonic represented in the boundary surface, and $Y_{pq}$ are fully normalized spherical harmonics of degree *p* and order *q*. We use the index *l* to indicate that this surface describes the outer boundary of the lower region. Care must be taken to select values for *χ* that prevent boundary surfaces from overlapping, so as to describe physically possible interior structures. Contour maps plotting the difference between layers are instructive for this purpose, as in Fig. 6, which was created with the analysis software we provide as Supplemental Material.

As we wish to describe physical surfaces, $r_{l}\left(\theta ,\phi \right)$ in Eq. (15) must be real-valued. The spherical harmonics we use are complex in general; so too must $\chi_{pq}^{l}$ be. Topographical descriptions in the literature against which we wish to compare are often described in terms of coefficients for real-valued, 4*π*-normalized spherical harmonics. The provided software includes a tool for converting this format into the coefficients $\chi_{pq}^{l}$, as we have used in our analysis. In Eq. (15), for ${\bar{r}}_{l}$ in meters, $\varepsilon_{l}$ is also in meters. For a perturbation represented by a pure harmonic of degree *p* and order *q*, $\varepsilon_{l}$ is the maximum radial displacement from a perfect sphere, and $\chi_{pq}^{l}$ is $1/Y_{pq}^{\max}$, where $Y_{pq}^{\max}$ is the value of $Y_{pq}$ at the location where $\left|Y_{pq}\right|$ is maximized.

Surfaces described by Eq. (15) are near-spherical in that we make the approximation $\varepsilon_{l}\ll {\bar{r}}_{l}$ for all *l*. Equivalently, $\varepsilon_{l}/\bar{r_{l}}\ll 1$, and we retain terms up to first order in $\varepsilon_{l}/\bar{r_{l}}$ only. This enables us to use a Taylor expansion in the boundary conditions that truncates quickly, adding only one term containing a product of two spherical harmonics. Products of spherical harmonics may be expressed as a linear combination of different harmonics (Wigner, 1931; Condon and Shortley, 1951). This results in “mixing” of harmonics in the excitation field from *n* = 1 into other *n*, so a uniform excitation field induces magnetic moments of quadrupole order or higher for this shape, in addition to altering the original induced dipole moments.

#### 2.4.1. Taylor expansion of boundary shapes

We account for asymmetry in the conductivity structure of the body by expanding the boundary surfaces about their average radii. To first order, a Taylor expansion of a function f(r) about $\bar{r_{l}}$ has terms

(16) $$f\left(r_{l}\right)\approx f\left({\bar{r}}_{l}\right)+\left(r_{l}-\bar{r_{l}}\right){\frac{\partial f\left(r\right)}{\partial r}|}_{r={\bar{r}}_{l}}=f\left({\bar{r}}_{l}\right)+\varepsilon_{l}\left[\sum_{p,q}\chi_{pq}^{l}Y_{pq}\left(\theta ,\phi \right)\right]{\frac{\partial f\left(r\right)}{\partial r}|}_{r={\bar{r}}_{l}}.$$

We evaluate these terms, then insert the result in place of the boundary radius in the general expressions for the field within each region (Eqs. S28–S33) in order to match the components of the field at the boundaries.

The next term in the expansion (the second-order term) will be proportional to $\varepsilon_{l}^{2}$ and the second derivative of f(r). Each derivative will contribute a division by *r* (from the Bessel functions; Marion and Heald, 1980), so each successive term gains another power of $\varepsilon_{l}/{\bar{r}}_{l}$, the ratio of the maximum deviation from spherical symmetry to the average radius of the boundary surface. We assume the boundaries are near-spherical, as $\varepsilon_{l}/{\bar{r}}_{l}$ is small—for Enceladus, for example, the maximum possible value is about 21 km/231 km ≈ 0.09 (Hemingway and Mittal, 2019). For Europa, the maximum ratio is even smaller, at about 30 km/1530 km ≈ 0.02 (Billings and Kattenhorn, 2005). The excitation moments experienced by icy moons in the solar system range from 7 nT for Triton up to about 300 nT for Miranda (Cochrane et al., 2021). The largest differences in magnetic field at the surface of these bodies from the first-order term can be about 10% for highly conducting, maximally asymmetric oceans. The second-order term can then only be about 1% of the maximum, up to a few nT on the surface. A lander on a highly asymmetric moon may need to account for second-order effects. However, at orbital/flyby altitudes and with realistic ocean shapes and conditions, we find that second-order effects are likely to be under 1 nT, and thus insignificant in a dynamic space environment.

## 3. Results

### 3.1. Analytic solution for the induced magnetic moments

Through application of the methods described above, for $B_{nm}^{i}$ of a body containing *N* conducting layers and an average outer radius *R*, we obtain a solution

(17) $$B_{nm}^{i}=\frac{n}{n+1}A_{n}^{e}B_{nm}^{e}+n\sum_{i=1}^{N}A_{n}^{t,i}K_{n}^{i}\Delta_{nm}^{i},$$

with

(18) $$\Delta_{nm}^{\text{i}}\equiv \{\begin{array}{ll} \sum_{n^{\prime },m^{\prime },p,q}\frac{\varepsilon_{\text{i}}\chi_{pq}^{\text{i}}}{{\bar{r}}_{\text{i}}}\Xi_{n^{\prime }m^{\prime }pq}^{⋆nm}\frac{{\bar{a}}_{nm}^{\text{i}}}{R}\left(j_{n^{\prime }}^{\text{i,i}}+\Lambda_{n^{\prime }}^{\text{i}}y_{n^{\prime }}^{\text{i,i}}\right)\left(k_{\text{i}}^{2}{\bar{r}}_{\text{i}}^{2}-k_{\text{i+1}}^{2}{\bar{r}}_{\text{i}}^{2}\right) & \text{for i}<N,\\ \sum_{n^{\prime },m^{\prime },p,q}\frac{\varepsilon_{N}\chi_{pq}^{N}}{R}\Xi_{n^{\prime }m^{\prime }pq}^{⋆nm}\frac{2n^{\prime }+1}{n^{\prime }+1}A_{n^{\prime }}^{⋆}B_{n^{\prime }m^{\prime }}^{e} & \text{for i}=N. \end{array}$$

Each quantity is fully determined by the interior structure model and the excitation moments, so the induced magnetic field can be directly calculated. $A_{n}^{e}$ is the complex amplitude for the zeroth-order (spherically symmetric) excitation, $A_{n}^{t,\text{i}}$ is the complex amplitude from the “output” harmonic *n, m* for the tangential component from the ith layer, and $A_{n^{\prime }}^{⋆}$ is the complex amplitude from the “input” harmonic that mixes into the output harmonic. As with $A_{n}^{e}$, the product $A_{n}^{t,N}A_{n^{\prime }}^{⋆}$ is asymptotic to (1 + 0*i*) in the limit $\left|k_{N}R\right|\to \infty$. The radial first-order term that would multiply a quantity $A^{r}$ analogous to $A^{t}$ is identically zero, so it does not appear. $\Delta_{nm}^{\text{i}}$ represents the strength of the induced magnetic field from each asymmetric boundary that “mixes” into the output harmonic *n, m* from the known excitation field. $K_{n}^{\text{i}}$ is a propagation factor that determines how the induced magnetic field resulting from a buried asymmetric boundary gets attenuated by the conducting layers above it. $\Xi_{n^{\prime }m^{\prime }pq}^{⋆nm}$ is a complicated function of Clebsch–Gordan coefficients that determines the strength of coupling between the given excitation moment $n^{\prime }$, $m^{\prime }$, a given boundary shape harmonic *p, q*, and the induced moment *n, m*. Please refer to the full derivation in Section S1 for more details, including definitions of every quantity.

Eq. (17) is our final result. Along with a suitable conductivity model and excitation moments from a magnetospheric model of the parent planet, this result may be applied to determine the induced magnetic field for a non-spherical ocean or ionosphere or both. Evaluating Eq. (17) takes only seconds on a modern desktop computer, offering a considerable advantage in run time over a numerical model. Benchmarking and comparison to a numerical model is a topic for future work.

### 3.2. Application to ocean worlds in our solar system

We now apply our model to several examples of icy moons in our solar system that are expected to contain asymmetry in their conductivity structure. We provide computer programs written in Python for evaluating our model as Supplemental Material, including the code for generating the plots in this Section. A full detailing of the results for each example, including contour maps of the asymmetry model and differences in the induced magnetic field arising from asymmetry, are contained in the Supplemental Text (Section S4). Here, we focus on the reasoning for the modeled asymmetry and the key findings from each application.

Conductivities within the ocean layers are determined using the *PlanetProfile* geophysical modeling framework (Vance et al., 2018, 2021). Several bulk parameters are used as inputs, such as surface temperature and moment of inertia, which are typically determined from spacecraft measurements; Table S1 lists each of the parameters we used. Ice shell thicknesses are assumed for each body. The interior structure is then determined self-consistently, accounting for solid-state convection in ice and adiabatic convection in the ocean, and using recently developed Gibbs-energy-based thermodynamics of solids and liquids. The ocean is assumed to be well-mixed, with a specified salt composition and concentration that is constant throughout the ocean. Depth-dependent pressures and temperatures are then combined with the salinity to determine ocean conductivities using the Gibbs Seawater model (McDougall and Barker, 2011) for Seawater or extrapolated from measurements reported by Larionov and Kryukov (1984) for MgSO_4_ oceans. Oceans are then reduced to three radial conducting layers before inserting them into our model, to reduce computational load. A greater number of layers may impact detailed analyses of spacecraft data, but the impact is expected to be small compared to other effects if ocean composition is uniform with depth (Vance et al., 2021). Asymmetry is then applied by describing the boundaries between radial layers as in Eq. (15). Ocean layers are assumed to be concentric, in that they each have the same asymmetric shape but scaled proportionally to the radius of the boundary.

Contour maps for Europa and Miranda show the asymmetry we model in the outermost ocean boundary, the ice–ocean interface for these bodies. The interiors for Callisto and Triton are assumed spherically symmetric; asymmetry is applied to their ionospheres based on a day–night dichotomy in ionization rate (Hartkorn et al., 2017). Interior contour maps may appear similar to those seen in the literature showing tidal flexure, but the similarity is only because they are each global maps portraying low-degree spherical harmonics. The difference from diurnal tides is relatively small (about 30 m for Europa; Moore and Schubert, 2000) so we assume static asymmetry in the ice–ocean interface. As the ionospheric asymmetry we assume for Callisto and Triton is due to day–night differences, the asymmetric shapes for these bodies will oscillate throughout their orbital periods (all the moons we study rotate synchronously). However, the strongest excitations applied to Callisto and Triton occur at their synodic periods with the parent planet’s rotation, 10–40 times more rapid than their orbital periods. For simplicity, we assume a fixed asymmetry in their ionospheres and consider our results an order-of-magnitude estimate for these bodies.

In all cases, the excitation moments applied to the moon are determined by taking a Fourier transform of the magnetic field at the position of the moon, evaluated over a 10-year time series. Body positions are determined using SPICE kernels available from NAIF.^4^,^5^. The magnetic field of the parent planet is calculated using a magnetosphere model from the literature: JRM09 + the Connerney current sheet model for Jupiter (Connerney et al., 2018, 1981), AH_5_ for Uranus (Herbert, 2009), and that of Connerney et al. (1991) for Neptune. Although we determine excitation moments for all significant periods, in this work we consider only the largest excitation, at the synodic period.

#### 3.2.1. Europa

**Fig. 1.**
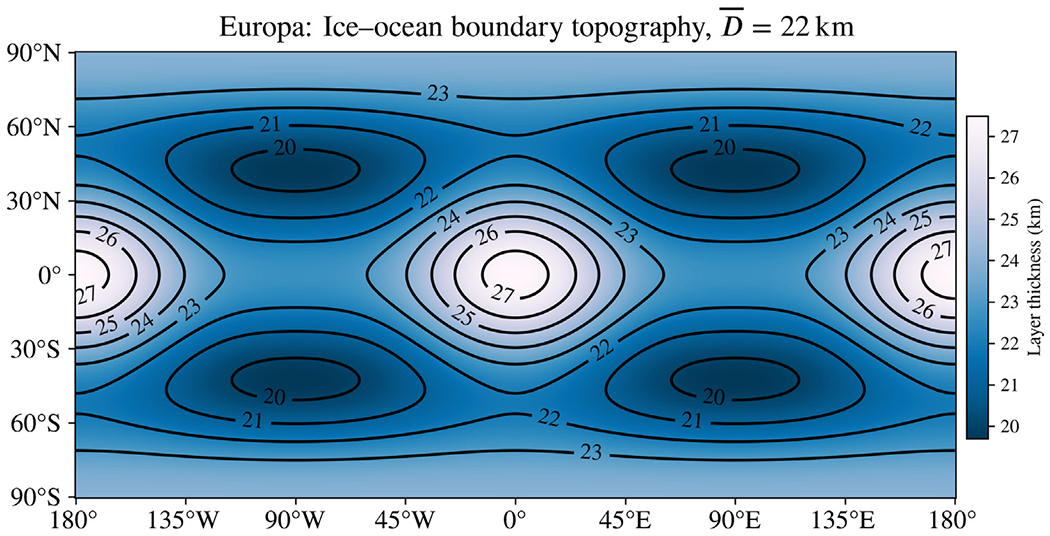
Asymmetry model for Europa, showing the thickness of the ice shell as a function of latitude and longitude in IAU coordinates, where (0°, 0°) is the sub-jovian point. The outer surface of Europa is assumed to be a perfect sphere and the ice–ocean boundary is perturbed. Then, both surfaces have tidal deformation added in accordance with *J*_2_ and *C*_22_ values reported by Anderson et al. (1998). We have supposed an ice shell asymmetry model approximating the results of Tobie et al. (2003); compare to Fig. 12a of that work. Ice thickness is 22.5 km on average and ranges from 20–27 km.

To model plausible asymmetry in the interior of Europa, we suppose a shape approximating the results of Tobie et al. (2003) for the ice shell thickness. In addition, we suppose a tidally deformed shape consistent with the static gravity coefficients *J*_2_ and *C*_22_ inferred from radio Doppler shifts due to acceleration of the *Galileo* spacecraft (Anderson et al., 1998)—see Section S3 for more details. Tobie et al. (2003) studied the dynamics of tidal heating and convection in Europa’s ice shell with numerical simulations, concluding that the shell is likely thickest at the sub- and anti-jovian points and thinnest around 60° latitude on the leading and trailing hemispheres. Fig. 1 shows the shape model we use to approximate this ice shell, which we have selected based on Fig. 12a of Tobie et al. (2003). We model the ocean using 3 concentric layers with the same boundary shape between layers, scaled to the radius of each boundary. A Seawater ocean is assumed; interior structure, including electrical conductivity, is evaluated using *PlanetProfile* as described above (Section 3.2). Conduction in the core is ignored for simplicity.

**Fig. 2.**
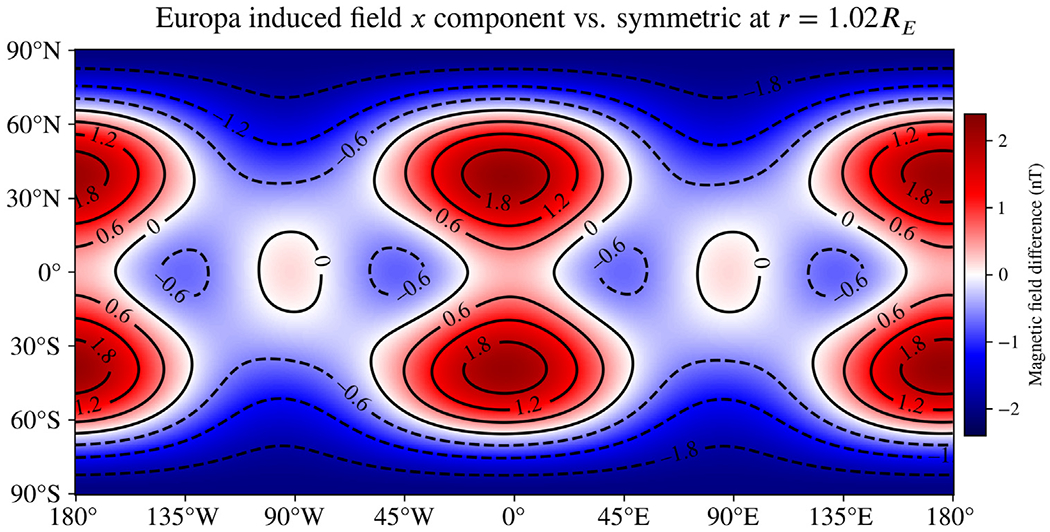
Difference in the *B*_*x*_ component (in IAU coordinates) of the induced magnetic field of Europa resulting from asymmetry in the ice–ocean boundary corresponding to the shape displayed in Fig. 1 and static gravity inferred by Anderson et al. (1998). A Seawater ocean composition is assumed. The magnetic fields are evaluated at the J2000 reference epoch and at 25 km altitude, consistent with the closest approach of several flybys planned by the *Europa Clipper* mission. This component of the induced field is changed by more than 1 nT in many locations, demonstrating that expected asymmetry in Europa’s ice shell is likely to have measurable effects in the nearest flybys by *Europa Clipper*. These closest flybys may help constrain the shape of Europa’s ice shell from magnetic measurements if precision reaches a few tenths of a nT. Tidal deformation is responsible for about 1/3 of the difference in this component. This global map of differences resulting from asymmetry changes throughout the synodic period; an animation showing the same map as it varies during the 11.2-hour period is included in the Supplemental Material.

Fig. 2 shows the difference in the *B*_*x*_ component (in IAU coordinates) of the induced magnetic field that results from the asymmetric structure shown in Fig. 1 at an altitude of 25 km. This altitude is chosen to assess the possible impact of our results on the *Europa Clipper* mission, which plans several flybys with closest approaches at this altitude or less (Campagnola et al., 2019). The displayed vector component is aligned with the strongest excitation applied by Jupiter; analogous maps for all components are shown in the Supplemental Material (Fig. S1). Differences of over 1.8 nT are observed in several locations at this time, the J2000 reference epoch. Static tidal deformation is responsible for about 1/3 of the difference in the *B*_*x*_ component (Fig. 2) and about half of the difference in the *B*_*z*_ component (Fig. S1f), which can be as much as 2.4 nT for the considered 11.2-hour synodic period. Although the background field of Jupiter is around 500 nT (Connerney et al., 2018), features of order 1 nT are routinely considered measurable in the analysis of spacecraft data (Horbury et al., 2020).

**Fig. 3.**
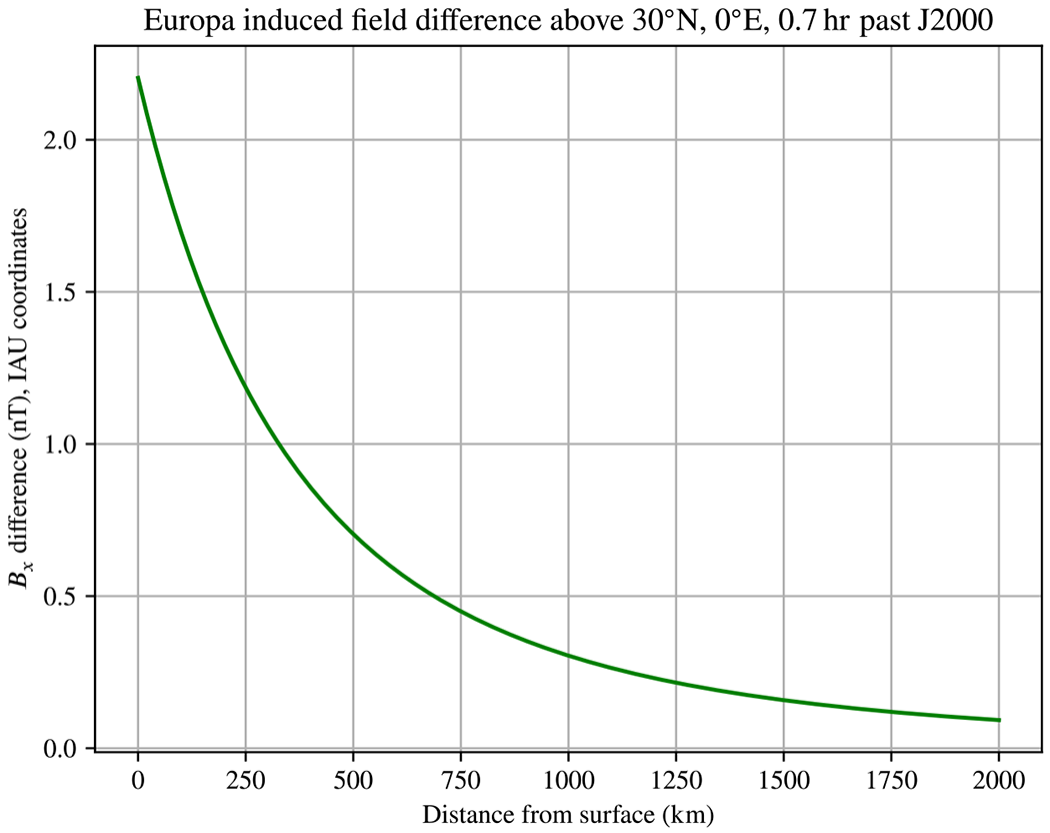
Difference in induced field *B*_*x*_ component for our Europa Seawater model as a function of altitude for a fixed point In time. The selected surface location (30°N, 0°E) and time (0.7 hr past J2000) maximize the observed difference relative to the spherically symmetric case for this Interior model and component—see Fig. 2. Beyond about 1500km altitude, the difference is around 0.2 nT and likely negligible.

The J2000 epoch is at an arbitrary point in time during Europa’s synodic period. At about 0.7 hr after J2000, the difference in *B*_*x*_ resulting from asymmetry is maximized. As seen in Fig. 2, the difference is also maximized near 30°N, 0°E. To form an initial estimate of how this asymmetric ocean model will impact spacecraft measurements at a variety of altitudes, we evaluated the induced field in a straight line normal to the surface above this location. Fig. 3 shows the difference resulting from asymmetry along this line. Near the surface, the difference is about 2.2 nT. The difference drops to 0.2 nT, likely small enough to ignore, only above an altitude of about 1500 km. Note also that the differences are sometimes small nearly everywhere—for example, the differences are below 0.6 nT at all points 25 km above the surface around *t* = 3.4 hr past J2000 (see Fig. S8).

**Fig. 4.**
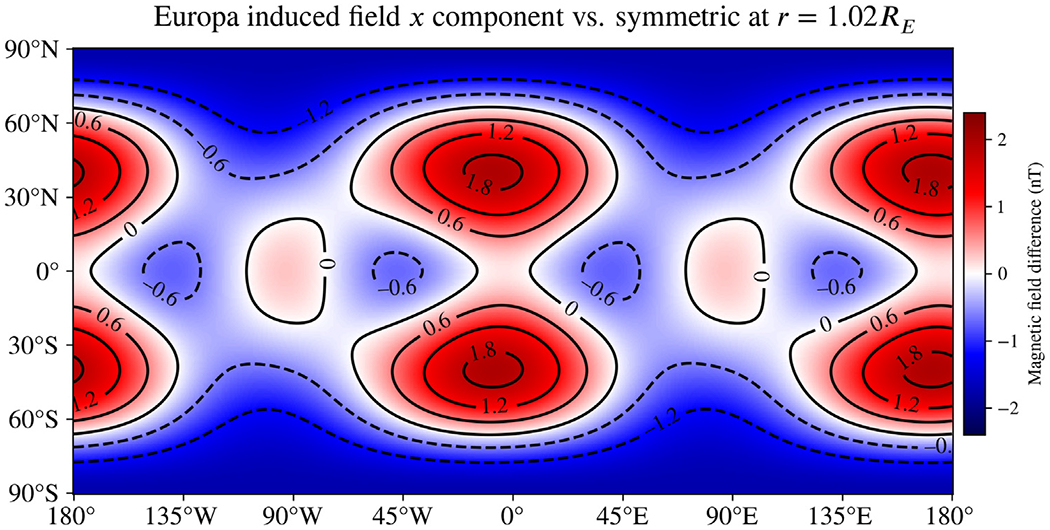
Difference in the *B*_*x*_ component of the induced magnetic field of Europa resulting from the asymmetry model shown in Fig. 1, as in Fig. 2, but with a salinity 10% that of Seawater. Comparison between the two cases shows these differences to be smaller for this component (60%–90% as great), as well as having a slight phase shift relative to the Seawater ocean. The colormap and contours for this plot are fixed to match those of Fig. 2.

We also evaluated a near-copy of this model, only with a salinity 10% that of Seawater, to examine the influence of asymmetry at other concentrations considered in the literature (Vance et al., 2021). The difference in the *B*_*x*_ component of the induced magnetic field resulting from asymmetry for this lower-salinity model is shown in Fig. 4. In this case, the differences are smaller by 10%–40% and a slight phase shift is observed. However, compared to the spherically symmetric analog, the differences are still as much as 2 nT. Europa’s relatively large size causes it to behave as a strong conductor and reject penetration by the time-varying excitation field with a strong induced field; this effect has a much stronger dependence on size than on conductivity or oscillation frequency (through an exponential dependence on $\left(r\sqrt{i\omega \mu \sigma }\right)$ in Bessel functions $j_{n}\left(kr\right)$, see Eq. (4)).

#### 3.2.2. Comparison with prior work

**Fig. 5.**
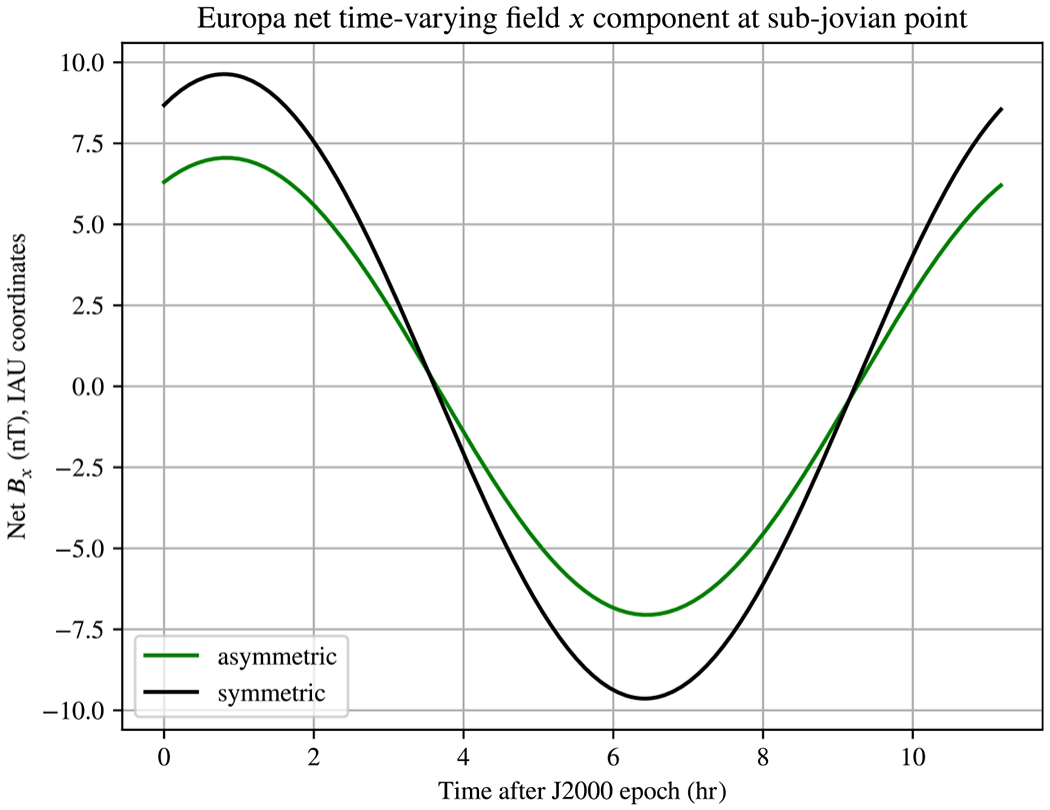
Vertical component of the net magnetic field a lander located at the sub-jovian point would measure on Europa for the asymmetric ice–ocean boundary studied in our previous work (Styczinski and Harnett, 2021), along with that of an analogous, spherically symmetric model. The ocean is treated as having effectively infinite conductivity, as in prior work. The effects of an ionosphere are neglected. The difference between the asymmetric and symmetric cases predicted by our complete model in this work is several times larger than the estimate from our previous work, about 2.5 nT at most. This model does not include tidal deformation.

In prior work (Styczinski and Harnett, 2021), we derived an approximation for evaluating the effects of asymmetry on induced fields, but in the limit of high conductivity in the conducting region (|kr|→∞). In this approximation, only the shape of the outermost boundary matters, because the time-varying excitation field is rapidly attenuated within the conducting material by the induced currents that generate the induced field. For comparison with that work, we now consider the exact same boundary shape studied in that work for Europa, *i.e.* by setting

(19) $$\begin{array}{ccc} \varepsilon_{l}=2.5\;\text{km}, & r_{l}=1537.5\;\text{km}, & \chi_{2,-2}^{l}=\chi_{2,2}^{l}=\frac{1}{2}\cdot 4\sqrt{\frac{2\pi }{15}} \end{array}$$

in Eq. (15) (note the difference in normalization of $\chi_{pq}^{l}$ in the earlier work). Fig. 5 shows the vertical component of the net magnetic field a lander at the sub-jovian point would measure throughout a synodic period, considering only that excitation period, analogous to Fig. 2 of Styczinski and Harnett (2021). In that work, the greatest difference was near the same points in time, though only about 0.5 nT.

Armed now with a rigorously derived, explicit formula for evaluating the mixing coefficients $\Xi_{n^{\prime }m^{\prime }pq}^{⋆\;nm}$ (Section S5), we find that the reason for the greater effect size compared to our previous work is that we had underestimated these coefficients. Our previous method for determining the values of $\Xi_{n^{\prime }m^{\prime }pq}^{⋆\;nm}$ did not account for non-orthogonality of the $Y_{nm}^{⋆}$ (see Section S1.5.3), and so scaled incorrectly when multiple excitation harmonics or multiple shape harmonics are represented. We now estimate a maximum difference of about 2.5 nT for this extreme limiting case, ignoring tidal deformation.

#### 3.2.3. Miranda

Miranda is the innermost large moon of Uranus. The orbital configuration of the large uranian moons implies past orbital resonances that likely caused tidal heating (Ćuk et al., 2020), and Miranda’s surface shows possible signs of past geologic activity (Beddingfield and Cartwright, 2020) that might have been provoked by this heating. The presence of ammonia-bearing compounds on the surface of nearby Ariel (Cartwright et al., 2020) implies lowered melting temperatures for the oceans on these moons (Croft et al., 1988), so liquid oceans may persist to the present even in this far-out system. With a radius of 235.8 km, Miranda is also comparable in size to Enceladus (radius 252.1 km). Enceladus has been found to have a marked asymmetry in its ice shell, with a notably thinner portion around the south pole (where plume activity is concentrated; Iess et al., 2014; Hemingway and Mittal, 2019). Together, these factors encourage a comparison between Miranda and Enceladus.

**Fig. 6.**
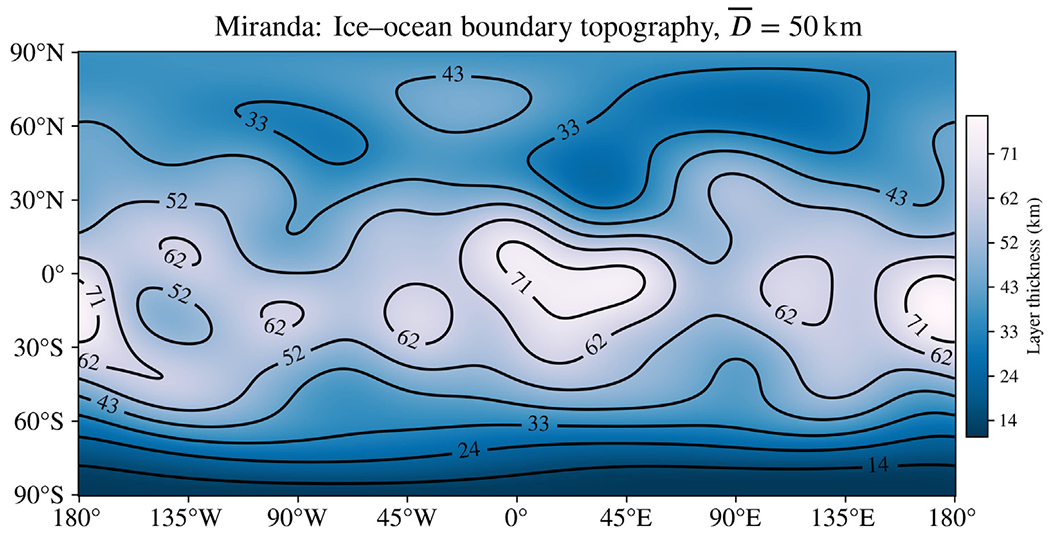
Possible “Enceladus-like” asymmetry in the ice shell of Miranda used in this study. The topography of the ice–ocean boundary for this body is scaled up from a model for Enceladus by Hemingway and Mittal (2019) inferred from isostatic compensation and spacecraft measurements. Compare to Fig. 11d from that work.

To model possible Enceladus-like asymmetry in the interior of Miranda, we scale the asymmetric topography of the ice–ocean boundary for Enceladus favored by Hemingway and Mittal (2019) based on isostatic compensation combined with gravity measurements and models of tidal heating in the ice shell. To account for the colder surface temperature of Miranda, which receives about 10% the insolation as Enceladus, we assume a 50 km thick ice shell, and scale the asymmetry model of Hemingway and Mittal (2019) from a 20 km average thickness to match. We consider this an upper estimate of asymmetry that may be possible within Miranda’s interior, as viscous relaxation is likely to smooth out the exaggerated features somewhat. The shape of the ice–ocean boundary model we have supposed for Miranda is shown in Fig. 6—compare to Fig. 11d of Hemingway and Mittal (2019). We assume an ocean composition of Seawater with 10% the salinity of Earth’s oceans, approximating the salinity of Enceladus inferred from *Cassini* Dust Analyzer sampling of the plumes (Postberg et al., 2009).

Miranda’s small size and proximity to Uranus prevent it from retaining a significant ionosphere. Comparison of the plasma environment (Mauk et al., 1987) to that near Callisto has led us to suppose a simple, uniformly conducting ionosphere that extends from the surface to 100 km altitude and has a total conductance of 800 S (Hartkorn et al., 2017) to serve as an upper limit. We assume a spherically symmetric ionosphere for Miranda; conduction in the ionosphere will serve only to attenuate the differences resulting from asymmetry as measured outside the body.

**Fig. 7.**
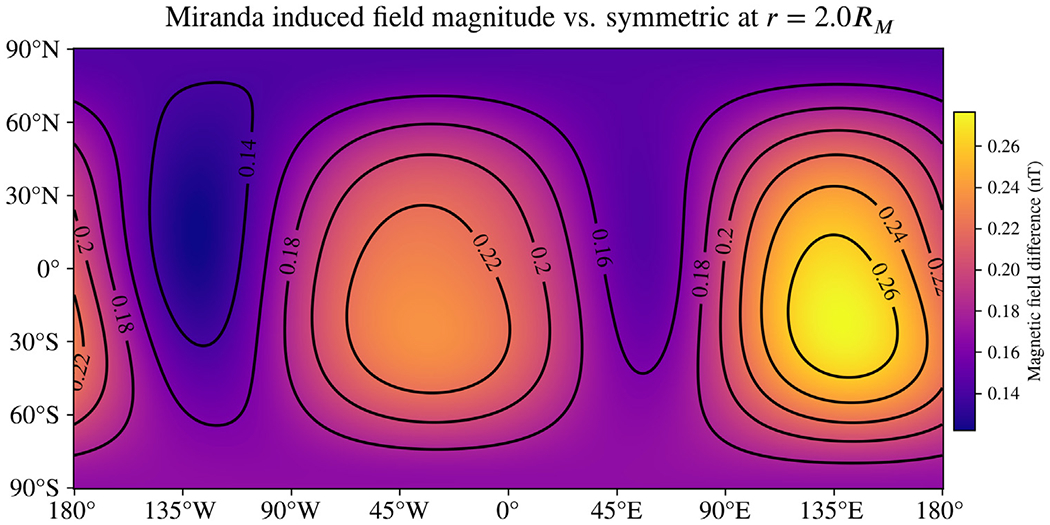
Difference in the magnitude of the induced magnetic field of Miranda resulting from the interior model shown in Fig. 6. Magnetic fields are evaluated at the J2000 reference epoch and at *r* = 2*R*_*M*_, a plausible flyby distance for a future mission. The simple geometry of the differences shown here reflects the fact that the change is dominated by increases in the dipole moments. This happens because the asymmetry model results in more conducting material residing closer to the surface, increasing the overall response to the excitation field. Unlike for Europa, the signal from asymmetry is over 20% of the total induced field in most places (compare to Fig. S4b). An animation showing the same map as it varies throughout the 35-hour synodic period is included in the Supplemental Material.

Fig. 7 shows the difference in the magnitude of the induced magnetic field resulting from the asymmetry model we have applied. The magnetic fields are again evaluated at the J2000 reference epoch, but this time at a distance *r* = 2*R*_*M*_, a plausible range around closest approach for flybys by a future Uranus orbiter. In this case, the differences are a significant fraction of the total induced field—over 20% in most places. As in other examples, all vector components are shown in the Supplemental Material (Fig. S4). Greater salinity in the ocean will result in larger differences resulting from asymmetry, though typically a smaller fraction of the total induced field. A closer approach will also increase the differences substantially, especially because of the quadrupole and octupole moments represented in the induced field, whose field strengths decrease faster than those of the dipole moments.

#### 3.2.4. Callisto and Triton

For both Callisto and Triton, we model spherically symmetric interiors and suppose asymmetry in their ionospheric structure based on a day–night dichotomy. This day–night difference occurs when the ionization rate is heavily influenced by EUV flux from the Sun, as is the expected case for Callisto (Hartkorn et al., 2017) and Triton (Krasnopolsky and Cruikshank, 1995). To approximate the asymmetric conductivity structure introduced by the day–night dichotomy, we apply a single degree-1 real harmonic for each moon, such that the uniformly conducting ionosphere bulges outward at local noon and inward at local midnight. Contour maps for these simple shapes are included in the Supplemental Text (Figs. S5a and S6a). Note that the asymmetric shape of the ionosphere will rotate in the frame of reference of the satellite on the same time scale as the orbital period. However, we aim to estimate the order of magnitude for the signal from asymmetry and demonstrate application of our model, so for simplicity we assume the asymmetric structure is fixed.

The ionosphere of Callisto is assumed to extend from the surface to 100 km altitude and to have a total conductance of 800 S, consistent with the estimate of Pedersen conductivity concluded by Hartkorn and Saur (2017). The upper boundary of this shape is then perturbed as described above. *Voyager 2* radio measurements revealed Triton to have a highly conductive ionosphere, with an ion density peaking well above the surface (Tyler et al., 1989). To model Triton’s ionosphere, we assume a uniformly conducting shell beginning at 250 km altitude and extending upward for 200 km. The upper boundary of this shape is then perturbed as described above, and the neutral atmosphere below is assumed to be nonconducting. In the highly conducting ionosphere of Triton, convection electric fields will tend to transport plasma from areas of high ion density to areas with lower density, smoothing out the asymmetry somewhat (Schunk and Nagy, 2009). To account for this, we decrease the amplitude of asymmetry compared to Callisto (to ±60 km rather than ±100 km).

Our conductivity models for both moons also contain conducting oceans in their interiors. For the interior of Callisto, we have assumed parameters consistent with previous studies of magnetic induction at this moon (*e.g.* Vance et al., 2021), namely a 100 km thick ice shell atop an ocean of 10 wt% MgSO_4_(aq). Magnetic induction at Triton has not yet been studied in detail; we have chosen the same composition for Triton’s ocean as for Callisto, and a slightly thicker ice shell of 112 km. Our Triton interior model is based on the moment of inertia and surface temperatures assumed for Pluto by Hussmann et al. (2006).

**Fig. 8.**
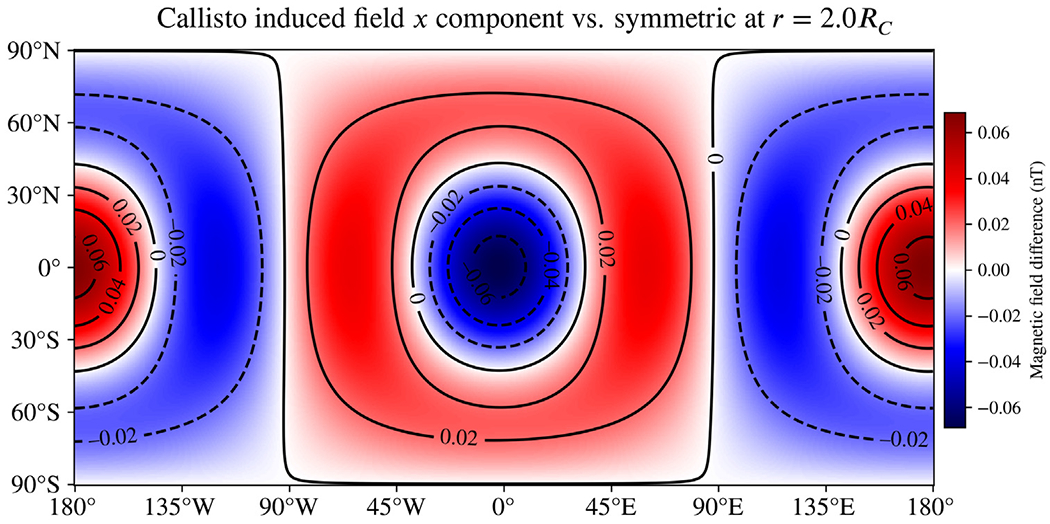
Difference in the *B*_*x*_ component of the induced magnetic field of Callisto resulting from asymmetry in the ionosphere representing a day–night dichotomy. Magnetic fields are evaluated at *r* = 2*R*_*C*_, consistent with a spacecraft flyby. The induced field is nearly unaffected by the large asymmetry in the ionosphere because of the small ionospheric conductivity (Hartkorn and Saur, 2017) and because the odd-degree shape harmonic we used does not cause any change to the dominant induced dipole moment.

**Fig. 9.**
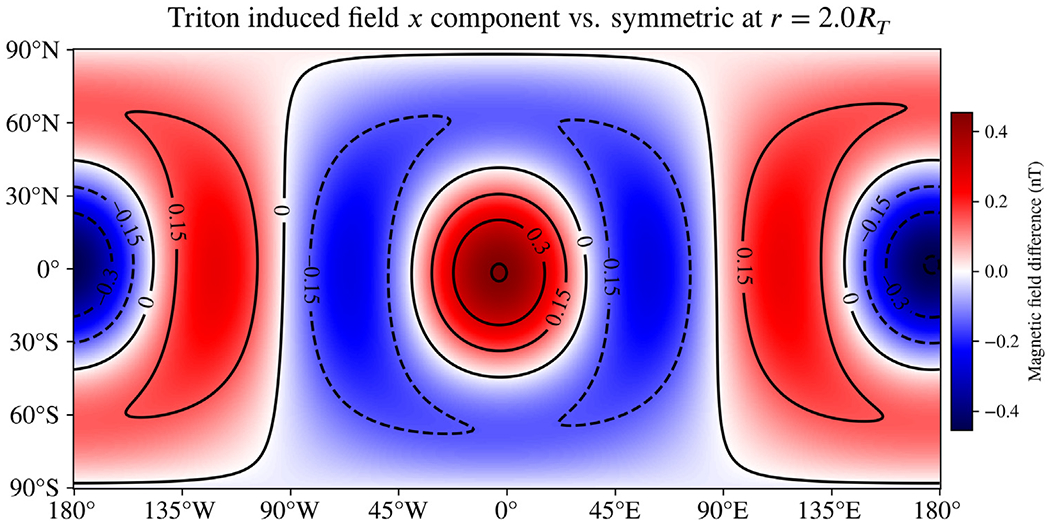
Difference in the *B*_*x*_ component of the induced magnetic field of Triton resulting from asymmetry in the ionosphere representing a day–night dichotomy. Magnetic fields are evaluated at *r* = 2*R*_*T*_, consistent with a flyby as part of a possible future mission. The high-altitude, highly conducting, asymmetric ionosphere we model introduces a significant difference relative to a spherically symmetric analog (about 1/3 of the total induced field, see Fig. S6b). This suggests ionospheric asymmetry is likely to play an important role in future magnetic investigations of Triton. However, the observed differences are limited to induced moments of quadrupole order and larger, as the asymmetric shape we use to represent the day–night dichotomy cannot change the induced dipole moments.

Figs. 8 and 9 show the differences in the *B*_*x*_ component of the induced magnetic field resulting from asymmetry in the ionospheres of Callisto and Triton, respectively. The magnetic fields are evaluated at the J2000 epoch and at *r* = 2*R*, where *R* is the body radius; as with Miranda, this distance is selected as a plausible flyby distance for past or future missions. Differences in the magnetic field from asymmetry in the Callisto ionosphere appear to be negligible, due primarily to the small ionospheric conductivity there. However, for Triton the high conductivity supports large differences, with the asymmetry accounting for as much as 1/3 of the total induced field (compare to Fig. S6b). For both moons, the degree-1 shape we use to perturb the upper ionospheric boundary is not capable of changing the induced dipole moment, so all differences will decay faster with distance than the main induction field from the spherically symmetric layers. Any asymmetry representing a day–night dichotomy will be dominated by odd-degree shapes describing the boundary due to their symmetry properties, so a more detailed asymmetric shape is unlikely to significantly change this result.

## 4. Discussion

The model we have developed is versatile in how it can be used to calculate induced magnetic fields. Our final result (Eq. (17)) is a direct generalization of the recursive method presented by Parkinson (1983) that is often used in magnetic sounding studies of icy moons (*e.g.* Schilling et al., 2007; Hand and Chyba, 2007; Seufert et al., 2011). Describing asymmetric boundaries between regions in terms of spherical harmonics, as in Eq. (15), permits a wide array of reasonable shapes to be supposed, allowing for forward models of the induced magnetic field of realistic, asymmetric oceans for the first time.

Our model takes only seconds to run, supporting Monte Carlo/Bayesian statistical methods that require a vast array of possible configurations in order to constrain interior properties. Such “big data” approaches are simply not possible with numerical solutions that require hours or days to evaluate. Furthermore, as our model determines the complex induced magnetic moments at a particular epoch, explicit time dependence is added simply with a factor *e^−iωt^* for each period of excitation. This allows our model to immediately be applied to evaluate the magnetic field at any date and time. Such functionality is already a part of the Python code we used to calculate results and create all figures, included as Supplemental Material.

### 4.1. Limitations of the analytic model

The necessary assumptions we have made in order to obtain an analytic result create some limitations to the applicability of the solution. The primary limiting assumptions are that of uniform conductivity within each conducting layer and that asymmetric boundaries are near-spherical.

#### 4.1.1. Uniform conductivity layers

Assuming uniform conductivity within each layer in the conducting body restricts our result to applications where the sequence of conducting layers along a line outward from the center is independent of latitude and longitude. In principle, it is possible to represent any closed surface containing the body center using a spherical harmonic expansion with high enough degree *p*. However, the advantages of taking such an approach with our model, *e.g.* to represent a melt lens within an ice shell, are likely outweighed by the additional complexity and computation time required. Higher-degree harmonics in such an expansion will have a much decreased weighting compared to low-degree harmonics, so the improved accuracy in the representation of the boundary will result in more modest gains in accuracy of representing the induced magnetic field. For these reasons, we recommend forming a low-degree (*p*_max_ ≤ 8) approximation for non-spherical boundaries to use with our model.

This condition requires that our solution is only approximate in cases where a rapid lateral change in conductivity is expected—for example, at the terminator line in Callisto’s ionosphere (Hartkorn et al., 2017). Far from the rapid lateral cutoff, our approach may still yield valid results, especially if the induced currents travel mostly parallel to the discontinuity. In the example of Callisto’s ionosphere, photoionization from solar EUV photons is the primary source of plasma, so a sharp dropoff in charge carriers, and hence conductivity, is expected behind the terminator line. To completely account for an abrupt lateral discontinuity in conductivity, a numerical solution is required. However, approximating a dichotomy like that in Callisto’s ionosphere with our approach still captures significant first-order effects of asymmetry and yields insights not possible to obtain with a spherically symmetric conductivity model.

#### 4.1.2. Near-spherical boundary shape

The second major assumption that limits applicability of our solution is that the boundaries between conducting regions are near-spherical. This assumption is required in order to limit our solution to a solvable set of equations describing a first-order change in the induced magnetic field. In other words, we are assuming that the second-order effects, *i.e.* the induced moments that result from the interaction of asymmetric boundaries with the new moments induced by the first-order expansion, are negligible. Each successive term in the Taylor expansion that determines the order of the solution (Eq. (16)) gains a new factor $\varepsilon_{l}/{\bar{r}}_{l}$ compared to the previous term. All other factors keep the same order of magnitude, although some mixing coefficients may grow or shrink slightly depending on the particular first-order induced moment and boundary shape considered. $\varepsilon_{l}/{\bar{r}}_{l}$ will generally be quite small for bodies large enough to differentiate, as their self-gravity is already sufficient to pull them into a near-spherical shape.

The value of the boundary deviation parameter $\varepsilon_{l}/{\bar{r}}_{l}$ for the bodies we studied ranges from 0.016 for Europa (Tobie et al., 2003, model) to 0.056 for the large Triton ionosphere, with the value for Miranda an outlier at 0.210. The asymmetry model for Enceladus (after Hemingway and Mittal, 2019) has a value of 0.071. We accounted for the thicker Miranda ice shell essentially by scaling up this parameter; our Miranda model serves to quantify an upper limit to the resulting differences from the spherically symmetric case for the assumed bulk properties. We found in Section 3.2.3 that the differences caused by this asymmetry were a significant fraction of the total induced field, but the total induced field was only on the order of 1 nT, so second-order effects would still be negligible in this case. If we had supposed a more saline ocean for Miranda, the induced field would grow substantially, and second-order effects could potentially become measurable. This may become a concern for future missions to the Uranus system, but we leave investigation of such second-order effects for future work.

### 4.2. Summary of example applications

Applying our analytic result to example moons as in Section 3.2, we provide evidence that the expected asymmetries are likely to result in measurable signals that will impact future magnetometer investigations. With the possible exception of Callisto, the conditions we studied resulted in significant differences compared to analogous spherically symmetric models: we find changes to the induced field of up to about 2.4 nT for Europa, 0.5 nT for Triton, and 0.25 nT for Miranda at plausible distances for orbiting spacecraft, and with marked variation in latitude, longitude, and time. In the case of asymmetric oceans, where degree-2 shape harmonics will be strongly represented, a significant amount of the change arises from alteration of the induced dipole moment. The size of this signal is likely to be significant for the upcoming *Europa Clipper* mission, which plans several near flybys of under 25 km. For the Europa Seawater model we studied, our initial estimates (Fig. 3) show that these effects will have measurable effects for a variety of flyby altitudes, but will also vary throughout the excitation period. However, we only considered this behavior for a single plausible model of asymmetry in Europa’s interior. It is not yet clear whether the effects of plausible asymmetry may be disregarded under some general condition, *e.g.* flybys above some threshold altitude. We recommend this as a topic for future work.

For Europa, we studied the same asymmetric shape under two different compositions, end-member Seawater oceans with salinities previously considered in the literature (*e.g.* Vance et al., 2021). From comparing these results (Figs. 2, 4), we can conclude that lowering the salinity had a relatively small impact on the signal arising from asymmetry. However, ocean composition is likely to play a bigger role at smaller moons such as Miranda and Enceladus than at Europa because the asymmetry there may be a significant fraction of radius. In addition, the smaller radii of the conducting boundaries render the predicted induced moments more sensitive to the electrical conductivity for the same reason Europa is less sensitive—an exponential dependence on $\left(r\sqrt{i\omega \mu \sigma }\right)$ in the Bessel functions $j_{n}\left(kr\right)$. The radii are large enough for Europa that ratios of these functions are close to unity, but the same does not apply to Miranda or Enceladus, thus causing changes in *σ* to have a greater effect on the induced field. The strong dependence on radius in these functions also causes asymmetric oceans that rise nearer to the surface to have outsize effects on the induced moments. This is evident in the differences in the magnitude of the induced field for Miranda in Fig. 7, which are universally positive for all latitudes and longitudes.

We did not consider magnetic fields arising due to currents in the plasma environment outside the bodies beyond the current sheet models used to determine the excitation moments. Such plasma fields will be critical to any future analysis of spacecraft measurements, but to include them in induction modeling is beyond the scope of this work.

## 5. Conclusions

With the mathematical methods described in this work, we have derived an approximate analytic model capable of calculating the induced magnetic field for any arbitrary planetary body to first order, provided it is near-spherical. This capability is an essential step forward in considering and accounting for the expected global asymmetries in ocean worlds. We have demonstrated that these asymmetries will produce measurable effects on the induced fields used in magnetic sounding investigations. These effects will introduce a source of systematic noise in measurements of order 2 nT near the surface of Europa, and will have a small impact on characterization studies using flyby data at all altitudes because the largest differences result from changes to the induced dipole moments. Future spacecraft investigations may be able to characterize asymmetry within the interiors of icy moons if they are able to achieve measurement precision on the order of a few tenths of a nT. Our analytic model and the software we provide for its evaluation support study of the interior structure of icy moons with an unprecedented level of detail.

## Supplementary Material

Supplemental text, computer code, and animations

## Footnotes

2 The meaning of “near-spherical” is clarified in Section 2.4—in short, this assumption should hold well in considering asymmetric oceans for most solar system moons. The consequences of our assumptions are elaborated in Section 4.1.

3 Also available as a Zenodo archive: https://zenodo.org/record/5002955

4 Generic SPICE kernels are hosted by NAIF at https://naif.jpl.nasa.gov/naif/data_generic.html

5 The specific kernels we used are pck00010.tpc and gm_de431.tpc for planetary constants, naif0012.tls for leap seconds, and jup310.bsp, ura111.bsp, and nep081.bsp for ephemerides.
