# Supplementary material for: A perturbation method for evaluating the magnetic field induced from an arbitrary, asymmetric ocean world analytically
Source: Icarus. Author manuscript; Available in PMC 2022 Apr 1. (PMC8819682; doi:10.1016/j.icarus.2021.114840)
Supplement: Supplemental text, computer code, and animations [file NIHMS1771783-supplement-Supplemental_text__computer_code__and_animations.zip › 1-s2.0-S0019103521004838-mmc1.pdf]

Supplemental Text for "A perturbation method for evaluating the magnetic field induced from an arbitrary, asymmetric ocean world analytically"

Marshall J. Styczinski[a,b,*], Steven D. Vance[c], Erika M. Harnett[b,d] and Corey J. Cochrane[c]

[a]Department of Physics, University of Washington, Box 351560, 3910 15th Ave NE, Seattle, WA 98195-1560, USA.
[b]UW Astrobiology Program, University of Washington, Box 351580, 3910 15th Ave NE, Seattle, WA 98195-1580, USA.
[c]Jet Propulsion Laboratory, California Institute of Technology, 4800 Oak Grove Dr, Pasadena, CA 91109-8001, USA.
[d]Department of Earth and Space Sciences, University of Washington, Box 351310, 4000 15th Ave NE, Seattle, WA 98195-1310, USA.

The final result from our derivation is complicated. To clarify the source of each piece of the result, we present each step of the derivation from first principles in Section S1. Python code for evaluating our model is provided as Supplemental Material[1]. Next, we present a table of values given as inputs to the *PlanetProfile* framework in Section S2 (Table S1). In Section S3, we give a detailed derivation of our method for translating J_2 and C_{22} gravity coefficients into geometric perturbations of boundary shapes. The full results from our example models applying our results for magnetic induction of asymmetric conducting layers in icy moons are presented in Section S4. Therein, we include figures showing the asymmetric layer topography, induced field magnitude at the considered altitude for the asymmetric model, and differences in the induced field components and magnitude that result from the asymmetry in the conductivity structure. Section S5 compiles explicit formulas for the Wigner $3j$-symbols and the mixing coefficients $\Xi^{nm}_{n'm'pq}$ and $\Xi^{\star\,nm}_{n'm'pq}$. Finally, in Section S6, we describe strategies for mitigating numerical difficulties with applying the layer method that result from the need to evaluate ratios of differences of very large, complex numbers. All figures appear at the end of the document.

S1. Derivation from first principles

Our goal is to apply Maxwell's laws at boundaries between regions and find the induced magnetic moments that satisfy the resulting equations. The same is true of the spherically symmetric, recursive solution developed by Srivastava (1966) and prominently applied by Zimmer et al. (2000), Seufert et al. (2011), and others. However, in our application, we describe the boundaries between regions of varying electrical conductivity by an arbitrary shape, rather than by a constant radius. The shape of the boundary has important consequences, and determines which magnetic moments are induced by a given excitation field applied by the parent planet. A derivation of the Srivastava recursive method is presented in more modern notation by the oft-cited Parkinson (1983). The Parkinson derivation contains several errors and inconsistencies, so we include here a full detailing of our solution from first principles so that we may best identify the consequences of those errors.

The excitation field applied to the conducting body has the form

$$\mathbf{B}_{\text{exc}}(\mathbf{r},t) = \mathbf{B}_o(\mathbf{r}) + \sum_{\text{j}} \mathbf{B}_{e,\text{j}}(\mathbf{r}) e^{-i\omega_{\text{j}} t}, \tag{S1}$$

with static ($\mathbf{B}_o$) and dynamic ($\mathbf{B}_{e,\text{j}}$) components that are complex in general. The measurable magnetic field is found by taking the real part of any complex expressions for the vector components. Superposition permits an independent handling of each excitation frequency, so we will focus on a single excitation frequency in our derivation. Our method may then be applied repeatedly for a combination of frequencies, and the results summed together.

*Corresponding author
mjstyczi@uw.edu (M.J. Styczinski); svance@jpl.caltech.edu (S.D. Vance); eharnett@uw.edu (E.M. Harnett); cjcochra@jpl.caltech.edu (C.J. Cochrane)
ORCID(s): 0000-0003-4048-5914 (M.J. Styczinski); 0000-0002-4242-3293 (S.D. Vance); 0000-0002-0025-7274 (E.M. Harnett); 0000-0002-4935-1472 (C.J. Cochrane)

[1]Also available as a Zenodo archive: https://zenodo.org/record/5002955.

In regions free of electric currents, the magnetic field satisfies Laplace's equation and therefore may be described by the gradient of a scalar potential ψ:

$$\nabla^2\mathbf{B} = 0 \tag{S2}$$
$$\mathbf{B} = -\nabla\psi. \tag{S3}$$

These equations are valid outside the conducting body (and outside any ionosphere) if we neglect currents in the magnetized plasma. Although currents in the plasma environment around the body are not generally negligible, the principle of superposition permits us to consider each contribution to the net electromagnetic response independently—the net magnetic field is then the sum from each individual contribution. In this work, we consider only the induced magnetic moments generated by the interaction of the primary excitation field with the conducting body, as this is the dominant interaction that induces magnetic fields from within solar system moons.

In spherical polar coordinates $\mathbf{r} = (r, \theta, \phi)$, general solutions to Eqs. S2 and S3 have the form (Jackson, 1999)

$$\psi(\mathbf{r}) = \sum_{n,m} R\left(B^e_{nm}\left(\frac{r}{R}\right)^n + B^i_{nm}\left(\frac{R}{r}\right)^{n+1}\right)Y_{nm}(\theta,\phi), \tag{S4}$$

where B^e_{nm} and B^i_{nm} are complex coefficients for the excitation and induced magnetic fields, Y_{nm} are spherical harmonics of degree n and order m, and R is a unit of radial distance, typically the outer radius of the conducting body. The B^e potentials, proportional to positive powers of r, can only be generated from outside of the conducting body under examination. The B^i terms in Eq. S4 are those of the multipole expansion, so each B^i_{nm} is proportional to, and thus represents, an induced multipole moment. We assume the magnetic potential for the excitation field oscillates sinusoidally, so time dependence is added to Eq. S4 by multiplication of $e^{-i\omega t}$ as in Eq. S1.

Within the conducting body, the dynamic excitation field induces electric fields that drive currents, so $\mathbf{B}$ cannot be represented by Eq. S3 in this region. Instead, we must use a diffusion equation for $\mathbf{B}$, derived from combining Maxwell's laws with Ohm's law:

$$\nabla^2\mathbf{B} = \mu\sigma\frac{\partial\mathbf{B}}{\partial t}. \tag{S5}$$

For simplicity in deriving our model, we neglect movement of conducting material within the body (as in the case of ocean currents) and rotation of the body within the external field, each of which can induce secondary fields (Saur et al., 2010; Vance et al., 2021). We also assume that the induced magnetic field is a linear phenomenon, *i.e.* the induction interaction is sufficiently weak that it does not affect the driving excitation field. As we are considering only the oscillatory magnetic field, taking the time derivative of $\mathbf{B}$ is equivalent to multiplication by $-i\omega$. We can thus rewrite Eq. S5 in terms of a diffusion constant k, and arrive at a vector Helmholtz equation:

$$\nabla^2\mathbf{B}_{\text{osc}} = -k^2\mathbf{B}_{\text{osc}} \tag{S6}$$
$$k = \sqrt{i\omega\mu\sigma}. \tag{S7}$$

The definition of k includes an arbitrary phase—we may also have chosen to include the $(-)$ underneath the square root, as in Parkinson (1983). This choice of phase will ultimately determine which differential equation we eventually find for the radial dependence of the internal magnetic field (Eq. S18).

In general, μ and σ are functions of position and will vary throughout the body. On planetary scales, μ is well approximated by μ_o, even for bodies containing large amounts of ferromagnetic materials (Saur et al., 2010); we assume $\mu = \mu_o$ everywhere in this work. We further assume that σ is a real-valued, uniform scalar within each conducting region (layer), and that each region is global in extent, *i.e.* any path outwards from the center must pass through each region sequentially.

General solutions to Eq. S6 for the configuration at hand must be consistent with a poloidal field. Toroidal fields can only exist within a source region, where poloidal currents are flowing (Moffatt, 1978; Backus, 1986; Backus et al., 1996), so no toroidal field can propagate from the parent planet to any moon through a nonconducting region, such as a hypothetically empty region of space, or a nonconducting ice shell. Toroidal fields can never be induced by an external source (Backus et al., 1996), no matter what shape the conducting body takes. In the source-free region, the magnetic field must satisfy the Laplace equation, which has solutions that yield poloidal fields, *e.g.* a uniform field as is common to model for that applied to a moon. The induced field must be able to oppose changes in magnetic flux due

to time variations in the driving field, and so must have a similar geometry to the driving field. However, the toroidal field is always perpendicular to the poloidal field and the radial direction (Backus, 1986), so a varying poloidal field can never induce a toroidal field.

Poloidal fields take the following form:

$$\mathbf{B}_\Psi = \nabla \times \nabla \times (\Psi \mathbf{r}) \tag{S8}$$

$$B_{r,\Psi} = \frac{1}{r}\left[-\frac{1}{\sin\theta}\frac{d}{d\theta}\sin\theta\frac{d}{d\theta} - \frac{1}{\sin^2\theta}\frac{d^2}{d\phi^2}\right]\Psi \tag{S9}$$

$$B_{\theta,\Psi} = \frac{1}{r}\frac{d}{d\theta}\frac{d}{dr}(\Psi r) \tag{S10}$$

$$B_{\phi,\Psi} = \frac{1}{r\sin\theta}\frac{d}{d\phi}\frac{d}{dr}(\Psi r), \tag{S11}$$

where the poloidal potential Ψ is a scalar function of position. Note that the expressions for the poloidal field vector components given by Parkinson (1983, Eq. 158) contain a sign error in the $\hat{r}$ component.

The quantity in square brackets in Eq. S9 is the angular momentum operator $\hat{L}^2$, of which the spherical harmonics Y_{nm} are eigenfunctions (Edmonds, 1996; Jackson, 1999):

$$\hat{L}^2 Y_{nm} = n(n+1)Y_{nm}. \tag{S12}$$

Note that Moffatt (1978, Eq. 2.26) presents Eq. S12 with a sign error based on their definition of the angular momentum operator. If we suppose Ψ is separable, we can expand it in spherical harmonics:

$$\Psi(r,\theta,\phi) = \sum_{n,m} c_{nm}\mathcal{R}_{nm}(r)\, Y_{nm}(\theta,\phi), \tag{S13}$$

where c_{nm} are constant coefficients determined by the boundary conditions and $\mathcal{R}_{nm}$ are functions we must determine from other relations. As we later satisfy the boundary conditions with this functional form of Ψ, the uniqueness theorem confirms that this is *the* physically correct representation (Dennery and Krzywicki, 2012), validating the supposition that Ψ is separable.

Inserting Eq. S13 into Eqs. S9–S11 and utilizing Eq. S12 yields expressions for the components of the magnetic field within the conducting body in terms of $\mathcal{R}$:

$$B_{r,\text{int}} = \sum_{n,m} \frac{c_{nm}}{r}\mathcal{R}_{nm}\, n(n+1)Y_{nm} \tag{S14}$$

$$B_{\theta,\text{int}} = \sum_{n,m} \frac{c_{nm}}{r}\frac{d}{dr}(r\mathcal{R}_{nm})\frac{\partial Y_{nm}}{\partial\theta} \tag{S15}$$

$$B_{\phi,\text{int}} = \sum_{n,m} \frac{c_{nm}}{r\sin\theta}\frac{d}{dr}(r\mathcal{R}_{nm})\frac{\partial Y_{nm}}{\partial\phi}. \tag{S16}$$

We can now make use of these expressions along with Eq. S6 to find a differential equation for $\mathcal{R}_{nm}$. Linearity of the ∇^2 operator allows us to consider only a single n, m term, as the same equations will apply to all terms. The $\hat{r}$ component of Eq. S6 reads as (Arfken et al., 2012)

$$\nabla^2 B_r - \frac{2B_r}{r^2} - \frac{2}{r^2\sin\theta}\frac{\partial}{\partial\theta}(\sin\theta B_\theta) - \frac{2}{r^2\sin\theta}\frac{\partial B_\phi}{\partial\phi} = -k^2 B_r. \tag{S17}$$

Inserting Eqs. S14–S16 and again exploiting the angular momentum operator, we arrive at a Bessel equation for $\mathcal{R}_{nm}$:

$$\frac{1}{\mathcal{R}_{nm}}\frac{d}{dr}\left(r^2\frac{d\mathcal{R}_{nm}}{dr}\right) + k^2r^2 - n(n+1) = 0. \tag{S18}$$

Solutions to this equation are linear combinations of the particular solutions, which are the spherical Bessel functions of the first and second kind, j_n and y_n:

$$\mathcal{R}_{nm}(r) = a_{nm}j_n(kr) + b_{nm}y_n(kr) \quad \text{or} \quad \mathcal{R}_{nm}(r) = a_{nm}\left(j_n(kr) + \Lambda_{nm}y_n(kr)\right) \text{ with } \Lambda_{nm} \equiv b_{nm}/a_{nm}, \tag{S19}$$

$$j_n(kr) = (-kr)^n \left(\frac{1}{kr} \frac{d}{d(kr)} \right)^n \frac{\sin kr}{kr}, \tag{S20}$$

$$y_n(kr) = -(-kr)^n \left(\frac{1}{kr} \frac{d}{d(kr)} \right)^n \frac{\cos kr}{kr}. \tag{S21}$$

a_{nm} and b_{nm} (or a_{nm} and Λ_{nm}) in Eq. S19 are constants determined from the boundary conditions; they will play a critical role in determining the induced magnetic field. The second format for $\mathcal{R}_{nm}$ presented in Eq. S19 is useful for expressing recursion relations for spherically symmetric bodies, but the first format must be used in solving the full boundary conditions for the asymmetric case. We will use both formats in our derivation.

S1.1. Consequences of the choice of phase for k

If, in defining the diffusion constant k in Eq. S7, we had chosen the alternate phase $k = \sqrt{-i\omega\mu\sigma}$, the first term in Eq. S18 would be negated. In that case, the resulting differential equation would be the *modified* spherical Bessel equation, with solutions $i_n(kr)$ and $k_n(kr)$, the *modified* spherical Bessel functions of the first and second kinds, respectively. These are similar to the spherical Bessel functions j_n and y_n and have similar properties; they are functions of $\sinh kr$ and $\cosh kr$ rather than $\sin kr$ and $\cos kr$. In the commonly cited derivation by Parkinson (1983, Ch. 5), this choice of phase for k should yield the modified spherical Bessel functions for the radial dependence of the internal magnetic field, but these authors incorrectly arrive at Eq. S18. Several authors (Zimmer et al., 2000; Khurana et al., 2002; Hand and Chyba, 2007; Arridge and Eggington, 2021) have repeated this error and applied a phase for k inconsistent with use of the standard Bessel functions. This error leaves the real part of the argument kr unchanged, while negating the imaginary part, equivalent to taking the complex conjugate.

The real part of kr determines the (real) exponential dependence of the Bessel functions, so the scale of the result is the same. The complex part determines the oscillation phase of the Bessel functions, and it becomes negated. In effect, the error described above causes the resulting solution for the induced magnetic field to *lead* the excitation field by the amount of the phase delay ϕ (Section 2.1.1), rather than lagging (as it must lag behind the excitation). Overall, the conclusions of the authors repeating this error are unaffected, but the expressions they apply all include a sign change in connecting the phase delay to the equations describing the induced magnetic field. For consistent comparisons to these important prior studies, we note by Eq. 8 (main text) that the phase delay ϕ is the *negative* of the phase of the complex amplitude $\mathcal{A}_1^e$ we later derive.

S1.2. General expressions for the magnetic field in each region

We must now use the expressions we have obtained to relate the magnetic field components at boundaries, as needed to solve Maxwell's equations. First, we note some considerations for the general radial dependence $\mathcal{R}_{nm}$ inside the conducting body: The solutions y_n diverge at the origin, so b_{nm} (or Λ_{nm}) must always be zero for the innermost region. As our solution for $\mathcal{R}_{nm}$ now contains arbitrary coefficients, we absorb the coefficients c_{nm} into a_{nm} and b_{nm}.

For later convenience, we also require expressions for $\frac{d}{dr}(r\,j_n(kr))$ and $\frac{d}{dr}(r\,y_n(kr))$. Eqs. S20 and S21 can be manipulated to obtain

$$j_n^\star \equiv \frac{d}{dr}(rj_n) = (n+1)j_n - kr\,j_{n+1}, \quad y_n^\star \equiv \frac{d}{dr}(ry_n) = (n+1)y_n - kr\,y_{n+1}, \tag{S22}$$

which we now also define as $j_n^\star$ and $y_n^\star$, respectively.

The details and formulation of the spherical harmonics are of central importance to this work. We use the fully normalized, complex spherical harmonics:

$$Y_{nm} = \sqrt{\frac{2n+1}{4\pi}\frac{(n-m)!}{(n+m)!}}\,P_n^m(\cos\theta)\,e^{im\phi}, \tag{S23}$$

$$Y_{n,-m} = (-1)^m Y_{nm}^*, \tag{S24}$$

where P_n^m are the associated Legendre functions with the Condon–Shortley phase. Although in the geomagnetics literature, the spherical harmonics are often expressed using real harmonics, in the Schmidt normalization, and without the Condon–Shortley phase, we use the fully normalized harmonics for optimal clarity in the derivation. Expressing each harmonic as succinctly as possible is vital, including their derivatives, products with other harmonics, and linear

combinations of harmonics. Each of these is made more complicated in the Schmidt normalization, so we use fully normalized harmonics to detail the mathematics. In the software we provide as supplemental material, we include an option to print the induced magnetic moments in the Schmidt normalization.

We will also later need expressions for θ derivatives of Y_{nm}:

$$Y^{\star}_{nm} \equiv \frac{\partial Y_{nm}}{\partial\theta} = \frac{1}{\sin\theta}\left(-w^{-}_{nm}Y_{n-1,m} + w^{+}_{nm}Y_{n+1,m}\right) \quad \text{for } m \geq 0, \tag{S25}$$

$$w^{-}_{nm} = (n+1)\sqrt{\frac{n^2-m^2}{(2n-1)(2n+1)}}, \quad w^{+}_{nm} = n\sqrt{\frac{(n+1)^2-m^2}{(2n+1)(2n+3)}}, \tag{S26}$$

$$Y^{\star}_{n,-m} = (-1)^m Y^{\star *}_{nm}. \tag{S27}$$

We thereby define $Y^{\star}_{nm}$ similar to $j^{\star}_n$ and $y^{\star}_n$, as they all pertain to the tangential components; we will not need the ϕ derivatives in our derivation. The format of Eq. S25, obtained using recurrence relations for the associated Legendre functions (*e.g.* Abramowitz and Stegun, 1972), has been selected for optimal use of orthogonality relations to solve the boundary conditions later.

We can now write general expressions for the magnetic field in all regions. From Eqs. S3 and S4, the external magnetic field follows

$$B_{r,\text{ext}} = \sum_{n,m}\left[-n\left(\frac{r}{R}\right)^{n-1}B^{e}_{nm} + (n+1)\left(\frac{R}{r}\right)^{n+2}B^{i}_{nm}\right]Y_{nm} \tag{S28}$$

$$B_{\theta,\text{ext}} = \sum_{n,m}\left[-\left(\frac{r}{R}\right)^{n-1}B^{e}_{nm} - \left(\frac{R}{r}\right)^{n+2}B^{i}_{nm}\right]Y^{\star}_{nm} \tag{S29}$$

$$B_{\phi,\text{ext}} = \sum_{n,m}\left[-\left(\frac{r}{R}\right)^{n-1}B^{e}_{nm} - \left(\frac{R}{r}\right)^{n+2}B^{i}_{nm}\right]\frac{1}{\sin\theta}\frac{\partial Y_{nm}}{\partial\phi}. \tag{S30}$$

From Eqs. S14–S16, the internal magnetic field follows

$$B_{r,\text{int}} = \sum_{n,m}\frac{a_{nm}j_n(kr) + b_{nm}y_n(kr)}{r}n(n+1)Y_{nm} \tag{S31}$$

$$B_{\theta,\text{int}} = \sum_{n,m}\frac{a_{nm}j^{\star}_n(kr) + b_{nm}y^{\star}_n(kr)}{r}Y^{\star}_{nm} \tag{S32}$$

$$B_{\phi,\text{int}} = \sum_{n,m}\frac{a_{nm}j^{\star}_n(kr) + b_{nm}y^{\star}_n(kr)}{r}\frac{1}{\sin\theta}\frac{\partial Y_{nm}}{\partial\phi}. \tag{S33}$$

The tangential components B_θ and B_ϕ offer redundant information in matching the solutions across the boundaries, so we will restrict our focus to the B_θ component because it contains terms for all n and m.

Solving for the a_{nm}, b_{nm}, and B^{i}_{nm} coefficients in these equations is accomplished by applying Maxwell's laws at the common boundaries between each region. On each boundary surface, Maxwell's laws dictate that the normal component of $\mathbf{B}$ must be continuous, and the tangential components of $\mathbf{B}/\mu$ must be continuous whenever there are no surface currents confined to the boundary itself (Jackson, 1999). As we assume $\mu = \mu_o$ within the body, $\mathbf{B}$ is continuous everywhere, and the components of the magnetic field for adjacent regions are equal on each boundary surface.

S1.3. Internal boundary conditions

At each boundary interior to the outer surface, the vector components of the magnetic field must match according to Eqs. S31–S33. At a distance from the body center $r_l(\theta,\phi)$ describing the outer boundary surface of a lower layer l with wavenumber k_l under an upper layer u with wavenumber k_u, the internal boundary conditions read

$$B_r: \quad \sum_{n,m}n(n+1)\frac{j_n(k_l r_l) + \Lambda^{l}_{nm}y_n(k_l r_l)}{r_l}a^{l}_{nm}Y_{nm} = \sum_{n,m}\frac{j_n(k_u r_l) + \Lambda^{u}_{nm}y_n(k_u r_l)}{r_l}a^{u}_{nm}Y_{nm}n(n+1) \tag{S34}$$

$$B_\theta\ : \qquad \sum_{n,m} \frac{j_n^\star(k_l r_l) + \Lambda_{nm}^l y_n^\star(k_l r_l)}{r_l} a_{nm}^l Y_{nm}^\star = \sum_{n,m} \frac{j_n^\star(k_u r_l) + \Lambda_{nm}^u y_n^\star(k_u r_l)}{r_l} a_{nm}^u Y_{nm}^\star. \tag{S35}$$

In general, r_l may be a function of θ and ϕ; this is the major focus of the present work. Formatting the linear combinations of j_n and y_n as we have done will, in the case of spherical symmetry, allow us to solve Eqs. S34 and S35 for Λ_{nm}^u in terms of Λ_{nm}^l, resulting in a recursion relation.

Mutual orthogonality of the spherical harmonics may be exploited to extract terms in Eq. S34 proportional to a desired harmonic $Y_{n'm'}$. Multiplying both sides by the complex conjugate of the desired harmonic $Y_{n'm'}^*$ and integrating over a unit sphere is equivalent to replacing $Y_{n'm'}$ with $\delta_{n,n'}\delta_{m,m'}$ (Kronecker delta functions), effectively discarding all other terms. A similar operation, multiplying both sides of Eq. S35 by $Y_{n'm'}^{\star *} \sin^2\theta$ and integrating over a unit sphere, yields somewhat different results but may be used to reach an analogous equation, as we now describe.

Linear combinations of orthogonal functions are not, in general, mutually orthogonal. From Eq. S25, we can determine that $Y_{nm}^\star \sin\theta$ may not be orthogonal to $Y_{n'm'}^\star \sin\theta$ when $m = m'$ and $n' = n + 2$ or $n' = n - 2$. One such example is $Y_{31}^\star$ and $Y_{11}^\star$: integrating $Y_{31}^{\star *} Y_{11}^\star \sin^2\theta$ over a unit sphere gives a non-zero result. This results from the overlap of the $Y_{n+1,m}$ term in Eq. S25 for $n = 1$ and the $Y_{n-1,m}$ term for $n = 3$. When this operation is applied in the sums over n and m in the boundary conditions, coupled linear equations result for these overlapping values of n. Ultimately, the equations are separable because of the following factors:

- For all terms, $n \geq 1$ because $\frac{\partial Y_{00}}{\partial\theta} = 0$.
- We can determine which a_{nm} are zero from the radial boundary condition equations, which have terms proportional to mutually orthogonal functions.
- The result of the overlap integral is the same in each conducting region, and so appears on both sides of each boundary condition equation.

The first two items above bound the number of equations. The final item may be used to scale and sum the equations so as to eliminate the terms proportional to $n' + 2$ or $n' - 2$, where the terms proportional to $Y_{n'm'}^\star$ are desired. The coefficient of the remaining n' term is altered by the overlap terms, but all terms are multiplied by the same coefficient (combinations of $w_{n'm'}^-$ and $w_{n'm'}^+$), so it divides away. Therefore, analogous to the radial equation, we finally obtain a result equivalent to replacing $Y_{nm}^\star$ by $\delta_{n,n'}\delta_{m,m'}$. These operations will be critical in collecting the new terms that arise from expanding the boundary radii in spherical harmonics.

S1.3.1. Spherically symmetric case

If we assume spherical symmetry in the boundary surface at r_l, Λ_{nm} in Eqs. S34 and S35 is independent of m and can be reduced using the orthogonality relations discussed above. Each value of m yields equations identical to other m, so we set $m = 0$ and drop that subscript on Λ. Multiplying both sides of Eq. S34 by $Y_{n'm'}^*$ and integrating over a unit sphere yields

$$a_{nm}^l \left(j_n(k_l r_l) + \Lambda_n^l y_n(k_l r_l)\right) = a_{nm}^u \left(j_n(k_u r_l) + \Lambda_n^u y_n(k_u r_l)\right) \tag{S36}$$

with $n = n'$. Multiplying both sides of Eq. S35 by $Y_{n'm'}^{\star *} \sin^2\theta$ and integrating over a unit sphere similarly yields

$$a_{nm}^l \left(j_n^\star(k_l r_l) + \Lambda_n^l y_n^\star(k_l r_l)\right) = a_{nm}^u \left(j_n^\star(k_u r_l) + \Lambda_n^u y_n^\star(k_u r_l)\right). \tag{S37}$$

Dividing these equations (S36 and S37) by each other, we can now solve for Λ_n^u in terms of Λ_n^l to obtain the desired recursion relations. The solution is

$$\Lambda_n^u = \frac{(j_n^{u,l} j_n^{\star l,l} - j_n^{l,l} j_n^{\star u,l}) + \Lambda_n^l (j_n^{u,l} y_n^{\star l,l} - y_n^{l,l} j_n^{\star u,l})}{(j_n^{l,l} y_n^{\star u,l} - y_n^{u,l} j_n^{\star l,l}) + \Lambda_n^l (y_n^{l,l} y_n^{\star u,l} - y_n^{u,l} y_n^{\star l,l})}, \tag{S38}$$

or

$$\Lambda_n^u = \frac{\delta_n^{u,l} + \Lambda_n^l \epsilon_n^{u,l}}{\beta_n^{u,l} + \Lambda_n^l \gamma_n^{u,l}} \tag{S39}$$

with

$$\alpha_n^{u,l} \equiv j_n^{u,l} y_n^{\star u,l} - y_n^{u,l} j_n^{\star u,l} = \frac{1}{k_u r_l} \tag{S40}$$

$$\beta_n^{u,l} \equiv j_n^{l,l} y_n^{\star u,l} - y_n^{u,l} j_n^{\star l,l} \tag{S41}$$

$$\gamma_n^{u,l} \equiv y_n^{l,l} y_n^{\star u,l} - y_n^{u,l} y_n^{\star l,l} \tag{S42}$$

$$\delta_n^{u,l} \equiv j_n^{u,l} j_n^{\star l,l} - j_n^{l,l} j_n^{\star u,l} \tag{S43}$$

$$\epsilon_n^{u,l} \equiv j_n^{u,l} y_n^{\star l,l} - y_n^{l,l} j_n^{\star u,l}, \tag{S44}$$

$$j_n^{l,l} \equiv j_n(k_l r_l), \quad y_n^{\star u,l} \equiv y_n^\star(k_u r_l), \quad etc. \tag{S45}$$

Although $\alpha_n^{u,l}$ does not appear in Eq. S39, it will later appear in the asymmetric solutions.

For N layers within the body, there are $N-1$ internal boundaries, so Eq. S39 must be applied $N-1$ times to obtain Λ_n^N. Recall that for the innermost layer, $\Lambda_n^1 = 0$, so the next layer above has $\Lambda_n^2 = \delta_n^{2,1}/\beta_n^{2,1}$. The notation in Eqs. S39–S44 is selected to be directly comparable to the recursion relations presented by Parkinson (1983) for the spherically symmetric case. However, Eq. S39 appears inverted because we have chosen Λ_n to be a coefficient for y_n instead of j_n. In Section S1.6, we will expand these results to first order about the boundary radius r_l to obtain our results for asymmetric boundaries.

S1.4. External boundary conditions

Combining Eqs. S28–S33, we obtain the boundary conditions that apply at the outermost $\left(N^{\text{th}}\right)$ conducting boundary of any shape:

$$B_r: \sum_{n,m} n(n+1) a_{nm}^N \frac{j_n(kr_N) + \Lambda_{nm}^N y_n(kr_N)}{r_N} Y_{nm} = \sum_{n,m} \left[-n \left(\frac{r_N}{R}\right)^{n-1} B_{nm}^e + (n+1) \left(\frac{R}{r_N}\right)^{n+2} B_{nm}^i \right] Y_{nm} \tag{S46}$$

$$B_\theta: \sum_{n,m} a_{nm}^N \frac{j_n^\star(kr_N) + \Lambda_{nm}^N y_n^\star(kr_N)}{r_N} Y_{nm}^\star = \sum_{n,m} \left[-\left(\frac{r_N}{R}\right)^{n-1} B_{nm}^e - \left(\frac{R}{r_N}\right)^{n+2} B_{nm}^i \right] Y_{nm}^\star, \tag{S47}$$

where $r_N(\theta,\phi)$ is the distance from the center of the body to the outermost conducting surface, with a nominal mean value of R. $k = k_N$ is that of the outermost conducting layer in the ocean. As with the internal boundary conditions, the only tangential component we consider is B_θ, as B_ϕ offers redundant information.

S1.4.1. Spherically symmetric case

In the case of spherical symmetry in the outer boundary surface, $r_N = R$. We can again exploit the orthogonality of the spherical harmonics to extract terms proportional to each individual Y_{nm} or $Y_{nm}^\star$. As described in Section S1.3.1, spherical symmetry in the interior layers results in all Λ_{nm} independent of m, but the same does not apply to the external coefficients if we wish to describe a general excitation field. Multiplying Eq. S46 by $Y_{n'm'}^*$ and inserting $r_N = R$, then integrating over a unit sphere we obtain

$$n(n+1)\frac{a_{nm}^N}{R}\left(j_n(kR) + \Lambda_n^N y_n(kR)\right) = -nB_{nm}^e + (n+1)B_{nm}^i \tag{S48}$$

with $n = n'$. Multiplying Eq. S47 by $Y_{n'm'}^{\star *} \sin^2\theta$ and inserting $r_N = R$, then integrating over a unit sphere we obtain

$$\frac{a_{nm}^N}{R}\left(j_n^\star(kR) + \Lambda_n^N y_n^\star(kR)\right) = -B_{nm}^e - B_{nm}^i, \tag{S49}$$

again with $n = n'$. If the interior boundaries are all spherically symmetric as well, Λ_n^N may be derived from the recursion relation (Eq. S39) using the desired interior layer model.

Eliminating a^N_{nm} as in the internal boundary conditions, we can solve for the unknown B^i_{nm} in terms of B^e_{nm}. In this spherically symmetric case, the solution is

$$B^i_{nm} = \frac{n}{n+1}\frac{\beta^N_n + \Lambda^N_n\gamma^N_n}{\delta^N_n + \Lambda^N_n\epsilon^N_n}B^e_{nm} \tag{S50}$$

with

$$\beta^N_n \equiv j^{\star N}_n - (n+1)j^N_n = -kRj_{n+1}(kR) \tag{S51}$$

$$\gamma^N_n \equiv y^{\star N}_n - (n+1)y^N_n = -kRy_{n+1}(kR) \tag{S52}$$

$$\delta^N_n \equiv nj^N_n + j^{\star N}_n = kRj_{n-1}(kR) \tag{S53}$$

$$\epsilon^N_n \equiv ny^N_n + y^{\star N}_n = kRy_{n-1}(kR), \tag{S54}$$

$$j^N_n \equiv j_n(kR), \quad etc. \tag{S55}$$

Our notation here differs slightly from that of Parkinson (1983), in that we factor out $n/(n+1)$ from the other terms in Eq. S50. This change allows us to readily make comparisons to the response of a perfectly conducting ocean. We collect the remaining parameters into another quantity, the complex response amplitude $\mathcal{A}^e_n$:

$$\mathcal{A}^e_n \equiv \frac{\beta^N_n + \Lambda^N_n\gamma^N_n}{\delta^N_n + \Lambda^N_n\epsilon^N_n} = -\frac{j_{n+1}(kR) + \Lambda^N_n y_{n+1}(kR)}{j_{n-1}(kR) + \Lambda^N_n y_{n-1}(kR)}, \tag{S56}$$

so that

$$B^i_{nm} = \frac{n}{n+1}\mathcal{A}^e_n B^e_{nm} \tag{S57}$$

describes the magnetic field induced by the body. Eq. S57 gives the primary response to the excitation field. The results of this work all represent perturbations to the spherically symmetric case; as a consequence, the complex response amplitude $\mathcal{A}^e_n$ appears in each result. At large $|kr|$, $\mathcal{A}^e_n$ is asymptotic to $(1 + 0i)$ for all n. $\mathcal{A}^e_n$ is independent of m, a result that will also hold in the asymmetric case, though the form of Eq. S57 will change.

In the commonly studied case of a uniform excitation field, with $n = 1$, $\mathcal{A}^e_n$ may be expressed in terms of the real amplitude A and phase delay ϕ (*e.g.* as defined by Zimmer et al., 2000):

$$\mathcal{A}^e_1 = Ae^{-i\phi}, \tag{S58}$$

allowing for ready comparison with prior work. Eq. S56 may therefore be used with a proposed layered structure model to easily calculate the response amplitude and lag phase in the case of spherical symmetry. Python programs we created for this purpose are provided as Supplemental Material.

Note that the negative exponent in Eq. S58 results from our definition of k in Eq. S7, as described further in Section S1.1. As a consequence of the error in the Parkinson (1983) derivation, both our input wavenumber k and our result for the complex response amplitude $\mathcal{A}$ are equal to the complex conjugate of the analogous quantities from prior work, hence the negative exponent in Eq. S58. We define the relationship between $\mathcal{A}^e_1$, A, and ϕ as in Eq. S58 to facilitate comparison with the rich set of prior work related to this topic.

S1.5. Near-spherical boundary shapes

We must now define surfaces $r_l(\theta,\phi)$ for near-spherical boundaries that we will insert into the internal and external boundary condition equations. Expanding each surface in spherical harmonics allows us to make use of relations well-known from problems involving addition of angular momenta from quantum mechanics, which we need because we will be multiplying harmonics together. We therefore choose boundary surfaces of the form

$$r_l(\theta,\phi) = \bar{r}_l + \varepsilon_l \sum_{p,q} \chi^l_{pq} Y_{pq}(\theta,\phi). \tag{S59}$$

$\bar{r}_l$ is the mean radius of boundary l, ε_l is the amplitude of deviation from spherical symmetry, χ^l_{pq} is a dimensionless constant that indicates the relative amount of each harmonic represented in the boundary surface, and Y_{pq} are fully

normalized spherical harmonics of degree p and order q. We use the index l to indicate that this surface describes the outer boundary of the lower region.

Surfaces described by Eq. S59 are near-spherical in that we make the approximation $\varepsilon_l \ll \bar{r}_l$ for all l. Equivalently, $\varepsilon_l/\bar{r}_l \ll 1$, and we retain terms up to first order in $\varepsilon_l/\bar{r}_l$ only. This approximation enables us to use a Taylor expansion in the boundary conditions that truncates quickly, adding only one term containing a product of two spherical harmonics. A product of spherical harmonics may be expressed as a linear combination of different harmonics (Wigner, 1931; Condon and Shortley, 1951). The multiplication of harmonics therefore results in "mixing" of harmonics in the excitation field from $n = 1$ into other n, so a uniform excitation field induces magnetic moments of quadrupole order or higher for this shape, in addition to altering the original dipole moments.

S1.5.1. Taylor expansion of boundary shapes

Let us now insert our near-spherical r_l into the expressions for the magnetic field in Eqs. S28–S33. To first order, a Taylor expansion of a function $f(r)$ about r_l has terms

$$f(r_l) \approx f(\bar{r}_l) + (r_l - \bar{r}_l)\frac{\partial f(r)}{\partial r}\Big|_{r=\bar{r}_l} = f(\bar{r}_l) + \varepsilon_l\Big[\sum_{p,q}\chi^l_{pq}Y_{pq}(\theta,\phi)\Big]\frac{\partial f(r)}{\partial r}\Big|_{r=\bar{r}_l}. \tag{S60}$$

The r^n power series that multiply B^e_{nm} and B^i_{nm} in Eqs. S28–S30 then have the form

$$r^n_l \approx \bar{r}^n_l\left(1 + n\frac{\varepsilon_l}{\bar{r}_l}\sum_{p,q}\chi^l_{pq}Y_{pq}\right). \tag{S61}$$

The interior field terms in Eqs. S31–S33 become

$$\begin{aligned}\frac{j_n(kr_l) + \Lambda^l_{nm}y_n(kr_l)}{r_l} &\approx \frac{1}{\bar{r}_l}\Bigg(j_n(k\bar{r}_l) + \Lambda^l_{nm}y_n(k\bar{r}_l)\\ &+\frac{\varepsilon_l}{\bar{r}_l}\Big[\sum_{p,q}\chi^l_{pq}Y_{pq}\Big]\Big[j^\star_n(k\bar{r}_l) + \Lambda^l_{nm}y^\star_n(k\bar{r}_l) - 2\big(j_n(k\bar{r}_l) + \Lambda^l_{nm}y_n(k\bar{r}_l)\big)\Big]\Bigg)\end{aligned} \tag{S62}$$

and

$$\begin{aligned}\frac{j^\star_n(kr_l) + \Lambda^l_{nm}y^\star_n(kr_l)}{r_l} &\approx \frac{1}{\bar{r}_l}\Bigg(j^\star_n(k\bar{r}_l) + \Lambda^l_{nm}y^\star_n(k\bar{r}_l)\\ &+\frac{\varepsilon_l}{\bar{r}_l}\Big[\sum_{p,q}\chi^l_{pq}Y_{pq}\Big]\Big[\big(j_n(k\bar{r}_l) + \Lambda^l_{nm}y_n(k\bar{r}_l)\big)\big(n(n+1) - k^2\bar{r}^2_l\big) - \big(j^\star_n(k\bar{r}_l) + \Lambda^l_{nm}y^\star_n(k\bar{r}_l)\big)\Big]\Bigg),\end{aligned} \tag{S63}$$

where k may correspond to that above or below the boundary (consistent throughout the expression), except at the outer boundary where all k take the value of the top conducting layer k_N. With the above expressions, we can now evaluate both the internal and external boundary conditions at the perturbed, near-spherical boundaries between layers.

S1.5.2. Products of spherical harmonics

The final piece required to express the internal and external boundary conditions in our method is to convert the products of spherical harmonics that result from the Taylor expansion into sums of other harmonics. We wish to express

$$Y_{nm}Y_{pq} = \sum_{n',m'}\Xi^{n'm'}_{nmpq}Y_{n'm'}, \tag{S64}$$

allowing us to replace these products where they appear so that we may retrieve the resulting magnetic moments through orthogonality of the spherical harmonics. Ξ are constant coefficients with subscripts indicating the input excitation harmonic n, m, the boundary shape harmonic p, q, and the output expansion harmonic n', m'. We also desire a similar expression for the analogous $\Xi^\star$ that will allow us to replace $Y^\star_{nm}Y_{pq}$, but it cannot be expressed as simply (see Section S1.5.3). Using fully normalized spherical harmonics Y that incorporate the Condon–Shortley phase, $\Xi^{n'm'}_{nmpq}$ are

the Clebsch–Gordan coefficients. Ultimately, we will multiply by $Y^*_{n'm'}$ or $Y^{\star *}_{n'm'}\sin^2\theta$ and integrate over a unit sphere; with this intent in mind, we will express Ξ in terms of the Wigner $3j$-symbols $\begin{pmatrix} j_1 & j_2 & J \\ m_1 & m_2 & M \end{pmatrix}$, which are proportional to the Clebsch–Gordan coefficients. The $3j$-symbols satisfy the useful identity

$$\int_0^{2\pi}\int_0^{\pi} Y_{nm}Y_{pq}Y_{n'm'}\sin\theta d\theta d\phi = \sqrt{\frac{(2n+1)(2p+1)(2n'+1)}{4\pi}}\begin{pmatrix} n & p & n' \\ 0 & 0 & 0 \end{pmatrix}\begin{pmatrix} n & p & n' \\ m & q & -m' \end{pmatrix} \tag{S65}$$

which follows from a related identity from Brink and Satchler (1968):

$$\int_0^{2\pi}\int_0^{\pi} (-1)^{m\delta_{m|m|}}P_{nm}e^{im\phi}\,(-1)^{m\delta_{q|q|}}P_{pq}e^{iq\phi}\,(-1)^{m\delta_{m'|m'|}}P_{n'm'}e^{im'\phi}\sin\theta d\theta d\phi = 4\pi\begin{pmatrix} n & p & n' \\ 0 & 0 & 0 \end{pmatrix}\begin{pmatrix} n & p & n' \\ m & q & m' \end{pmatrix}, \tag{S66}$$

where P_{nm} are Schmidt semi-normalized associated Legendre functions without the Condon–Shortley phase. The general expression for the Wigner $3j$-symbols is given in Section S5. The selection rules, which dictate the combinations of harmonics yielding non-zero terms, are summarized as follows (Brink and Satchler, 1968):

- $|n-p| \le n' \le n+p$, often called the "triangular condition."
- $m' = -(m+q)$. The general expression for the $3j$-symbols is unchanged under $m' \to -m'$ (see Eq. S97). From Eq. S24, we see that Eq. S65 is unchanged if we replace $Y_{n'm'}$ with $(-1)^{m'}Y^*_{n',-m'}$. We therefore use the selection rule $m' = m+q$ for clarity in our derivation and negate m' in the final $3j$-symbol, as we have done in Eq. S65.
- When $m = q = m' = 0$, the $3j$-symbols are non-zero only if $n+p+n'$ is even. Therefore, only $n' = n+p,\ n+p-2,\ n+p-4,\ \dots\ |n-p|$ give nonzero terms.

The results of the integration in each of Eqs. S65 and S66 are proportional to $\begin{pmatrix} n & p & n' \\ 0 & 0 & 0 \end{pmatrix}$, so all products are subject to all of these conditions.

Finally, we now obtain a direct expression for the product coefficients $\Xi^{n'm'}_{nmpq}$ to replace the products $Y_{nm}Y_{pq}$ in the radial boundary conditions. Multiplying both sides of Eq. S64 by $Y^*_{n'm'}$ and integrating over a unit sphere, we obtain

$$\Xi^{n'm'}_{nmpq} = (-1)^{m'}\sqrt{\frac{(2n+1)(2p+1)(2n'+1)}{4\pi}}\begin{pmatrix} n & p & n' \\ 0 & 0 & 0 \end{pmatrix}\begin{pmatrix} n & p & n' \\ m & q & -m' \end{pmatrix} \tag{S67}$$

$$\text{for}\quad |n-p| \le n' \le n+p,\quad n+p+n'\ \text{is even},\quad \text{and}\quad m' = m+q,$$

$$\Xi^{n'm'}_{nmpq} = 0 \quad \text{otherwise.} \tag{S68}$$

Using the selection rules and direct expressions for the Wigner $3j$-symbols (Eqs. S97 and S98), we can derive an explicit formula for the nonzero terms in $\Xi^{n'm'}_{nmpq}$ (see Eq. S101):

$$\Xi^{n'm'}_{nmpq} = (-1)^{\nu}\sqrt{\frac{(2n+1)(2p+1)(2n'+1)\,(n'+m')!(n'-m')!}{4\pi\,(n+m)!(n-m)!(p+q)!(p-q)!}}\,\frac{(n+p-\nu)!}{\nu!(n-\nu)!(p-\nu)!}\quad\times \tag{S69}$$

$$\frac{(2n-2\nu)!(2p-2\nu)!}{(2n'+1+2\nu)!}\sum_{\kappa=\kappa^-}^{\kappa^+}\frac{(-1)^{\kappa}(2\nu)!}{\kappa!(2\nu-\kappa)!}\,\frac{(n+m)!(n-m)!(p+q)!(p-q)!}{\big(n+m-(2\nu-\kappa)\big)!\big(n-m-\kappa\big)!\big(p+q-\kappa\big)!\big(p-q-(2\nu-\kappa)\big)!}$$

for $\nu = \frac{1}{2}(n+p-n')$, $\kappa^- = \max(0,\ 2\nu-(n+m),\ 2\nu-(p-q))$, and $\kappa^+ = \min(2\nu,\ n-m,\ p+q)$. These expressions allow us to use the replacement rule $Y_{nm}Y_{pq} \to \sum_{n',m',p,q}\Xi^{n'm'}_{nmpq}$.

We also require a similar expression that allows us to make the same replacement for the products $Y^\star_{nm}Y_{pq}$ in the tangential boundary conditions. Although we are able to make this replacement owing to separability of the linear equations, lack of orthogonality in $Y^\star_{nm}$ results in an extremely complicated expression for $\Xi^{\star n'm'}_{nmpq}$. A finite expression may nevertheless be obtained under the condition that we assume a maximum n in the excitation field $n_{\max}$ and a maximum p in the boundary shape $p_{\max}$. For $n > n_{\max}$, $B^e_{nm} = 0$ and for $p > p_{\max}$, $\chi^l_{pq} = 0$, so the sums over these indices truncate.

From Eqs. S25, S65, and S67, we obtain

$$\int_0^{2\pi}\int_0^{\pi} Y^\star_{nm} Y_{pq} Y^{\star *}_{n'm'} \sin^3\theta d\theta d\phi = \Xi^w_{n'}, \tag{S70}$$

$$\Xi^w_{n'} \equiv w^-_{nm} w^-_{n'm'} \Xi^{n'-1\,m'}_{n-1\,mpq} + w^+_{nm} w^+_{n'm'} \Xi^{n'+1\,m'}_{n+1\,mpq} - w^-_{nm} w^+_{n'm'} \Xi^{n'+1\,m'}_{n-1\,mpq} - w^+_{nm} w^-_{n'm'} \Xi^{n'-1\,m'}_{n+1\,mpq}, \tag{S71}$$

where $w^\pm_{nm}$ are defined in Eq. S26. We have defined $\Xi^w_{n'}$ for compactness of notation; n' is the only index which varies for this quantity in subsequent expressions, although all other indices n, m, p, q, m' are implied and required to evaluate it. This quantity roughly represents the amount of overlap between the product $Y^\star_{nm} Y_{pq}$ and the desired harmonic $Y^\star_{n'm'}$.

To next express $\Xi^{\star\,n'm'}_{nmpq}$ compactly, we must also define the following quantities, which are continued fractions:

$$F^\pm_{n,2\kappa} \equiv \frac{w^\pm_{n\pm 2\kappa} w^\mp_{n\pm(2\kappa+2)}}{\left(w^-_{n\pm(2\kappa+2)}\right)^2 + \left(w^+_{n\pm(2\kappa+2)}\right)^2 - w^\pm_{n\pm(2\kappa+2)} w^\mp_{n\pm(2\kappa+4)} F^\pm_{n,2\kappa+2}}, \tag{S72}$$

where $2\kappa = 0, 2, 4, \ldots$ and a subscript m is implied on all w. The recursion in $F^-_{n,2\kappa}$ continues until 2κ exceeds $n - |m| - 2$ and the recursion in $F^+_{n,2\kappa}$ continues until 2κ exceeds $n_{\max} + p_{\max} - n$. When 2κ exceeds these respective values, $F^\pm_{n,2\kappa} = 0$. $F^-_{n,2\kappa}$ is also zero for $n < 3$ because there are no $Y^\star_{n'm'}$ below $n = 2$ that can mix into $n = 2$, and the same is true for $n = 1$. These bounding values result from non-orthogonality of the $Y^\star_{nm}$. For a term of degree n'' below the considered value n', non-orthogonality is possible only for $n'' = n' - 2$, $n' - 4$, *etc.*, but $Y^\star_{n''m''}$ is defined and non-zero only for $n'' > 0$ and $n'' \geq m''$. This constraint limits the non-zero values of $F^-_{n',0}$ to $n' \geq 3$ and $n' \geq m' + 2$. The n'' above the considered n' that yield non-orthogonal terms are similarly limited by the selection rules for $\Xi^{n'm'}_{nmpq}$, *i.e.* n'' cannot exceed $n_{\max} + p_{\max}$ because no combination of $Y^\star_{nm} Y_{pq} \to \Xi^{\star\,n'm'}_{nmpq} Y^\star_{n'm'}$ results in $n' > n_{\max} + p_{\max}$ and we have assumed the series in n' may be truncated at $n'_{\max} = n_{\max} + p_{\max}$.

We obtain solvable linear equations by multiplying both sides of the tangential boundary condition equations (Eqs. S35 and S47) by $Y^{\star *}_{n'm'} \sin^2\theta$, then integrating over a unit sphere. Solving the resulting equations for the terms initially proportional to $Y^\star_{n'm'}$, we finally obtain the replacement rule $Y^\star_{nm} Y_{pq} \to \sum_{n',m',p,q} \Xi^{\star\,n'm'}_{nmpq}$, with

$$\Xi^{\star\,n'm'}_{nmpq} = \frac{\Xi^w_{n'} + \sum_{\nu=1}^{g^-_{\max}} \Xi^w_{n'-2\nu} \prod_{\kappa=0}^{\nu-1} F^-_{n',2\kappa} + \sum_{\nu=1}^{g^+_{\max}} \Xi^w_{n'+2\nu} \prod_{\kappa=0}^{\nu-1} F^+_{n',2\kappa}}{\left(w^-_{n'}\right)^2 + \left(w^+_{n'}\right)^2 - w^-_{n'} w^+_{n'-2} F^-_{n',0} - w^+_{n'} w^-_{n'+2} F^+_{n',0}}, \tag{S73}$$

$$g^-_{\max} \equiv \begin{cases} \text{floor}\left(\frac{1}{2}\left(n' - |m'|\right)\right) & \text{if } n' \geq 3 \\ 0 & \text{otherwise} \end{cases}, \quad g^+_{\max} \equiv \text{floor}\left(\frac{1}{2}\left(n_{\max} + p_{\max} - n'\right)\right), \tag{S74}$$

where floor(a) is the nearest integer less than or equal to a.

S1.5.3. Consequences of non-orthogonality of $Y^\star_{nm}$

In our previous work (Styczinski and Harnett, 2021), based on several considered cases we assumed that certain linear combinations of the $Y^\star_{nm}$ could be made mutually orthogonal. Although this assumption seems to be true when the shape harmonics Y_{pq} are limited to $p_{\max} = 2$, in this work we have endeavored to find a more general solution that may be applied to all $Y^\star_{nm}$ and for any $p_{\max}$. The primary consequence of non-orthogonality of the $Y^\star_{nm}$ is coupling between the boundary condition equations. This coupling is why $\Xi^{\star\,n'm'}_{nmpq}$ is not equal to $\Xi^w_{n'}$, and may be non-zero for any allowed n' and m' that differ from $(n + p)$ by an even integer. The end result is that some harmonics in the near-spherical boundaries can produce induced moments with high degree n'. For example, for a boundary surface containing harmonics up to degree $p_{\max} = 8$, as we consider in our Miranda model in Section 3.2.3, the Y_{21} boundary harmonic combines with the Y_{11} excitation harmonic to produce induced moments of degree $n' = 1, 3, 5, 7$, and 9. In this work, though we calculate all of the induced magnetic moments, we only plot the magnetic fields for n' up to 4, as the induced moments are already negligible at that degree, and the fields from these moments also shrink faster with distance from the body. Although the induced fields from the high-degree moments are likely to be small at spacecraft distances, they are nevertheless predicted by our model, a direct result from the non-orthogonality of the $Y^\star_{nm}$.

S1.6. Boundary conditions with a near-spherical boundary shape

Solving all the boundary conditions with near-spherical boundaries relies on simultaneous Taylor expansions of each boundary. For clarity in the end solution, we will now exchange the arbitrary indices n and n', so that n' refers to the "input" harmonics pertaining to the excitation field and n refers to the "output" harmonics that index the induced magnetic moments. We must also now use $b_{nm} = a_{nm}\Lambda_{nm}$ for the asymmetric layer coefficients, as a solution cannot be obtained otherwise. The procedures described in Section S1.5 result in the following boundary conditions for asymmetric bodies:

$$B_{r,\mathrm{int}}: \tag{S75}$$
$$n(n+1)\left(a^l_{nm}j^{l,l}_n + b^l_{nm}y^{l,l}_n\right) + \sum_{n',m',p,q}\frac{\varepsilon_l\chi^l_{pq}}{\bar{r}_l}\Xi^{nm}_{n'm'pq}\,n'(n'+1)a^l_{n'm'}\left((j^{\star l,l}_{n'} + \Lambda^l_{n'}y^{\star l,l}_{n'}) - 2(j^{l,l}_{n'} + \Lambda^l_{n'}y^{l,l}_{n'})\right) =$$
$$n(n+1)\left(a^u_{nm}j^{u,l}_n + b^u_{nm}y^{u,l}_n\right) + \sum_{n',m',p,q}\frac{\varepsilon_l\chi^l_{pq}}{\bar{r}_l}\Xi^{nm}_{n'm'pq}\,n'(n'+1)a^u_{n'm'}\left((j^{\star u,l}_{n'} + \Lambda^u_{n'}y^{\star u,l}_{n'}) - 2(j^{u,l}_{n'} + \Lambda^u_{n'}y^{u,l}_{n'})\right)$$

$$B_{\theta,\mathrm{int}}: \tag{S76}$$
$$a^l_{nm}j^{\star l,l}_n + b^l_{nm}y^{\star l,l}_n + \sum_{n',m',p,q}\frac{\varepsilon_l\chi^l_{pq}}{\bar{r}_l}\Xi^{\star\,nm}_{n'm'pq}\,a^l_{n'm'}\left((n'(n'+1) - k_l^2\bar{r}_l^2)(j^{l,l}_{n'} + \Lambda^l_{n'}y^{l,l}_{n'}) - (j^{\star l,l}_{n'} + \Lambda^l_{n'}y^{\star l,l}_{n'})\right) =$$
$$a^u_{nm}j^{\star u,l}_n + b^u_{nm}y^{\star u,l}_n + \sum_{n',m',p,q}\frac{\varepsilon_l\chi^l_{pq}}{\bar{r}_l}\Xi^{\star\,nm}_{n'm'pq}\,a^u_{n'm'}\left((n'(n'+1) - k_u^2\bar{r}_l^2)(j^{u,l}_{n'} + \Lambda^u_{n'}y^{u,l}_{n'}) - (j^{\star u,l}_{n'} + \Lambda^u_{n'}y^{\star u,l}_{n'})\right)$$

$$B_{r,\mathrm{ext}}: \tag{S77}$$
$$\frac{n(n+1)}{R}\left(a^N_{nm}j^N_n + b^N_{nm}y^N_n\right) + \sum_{n',m',p,q}\frac{\varepsilon_N\chi^N_{pq}}{R}\Xi^{nm}_{n'm'pq}\frac{n'(n'+1)}{R}a^N_{n'm'}\left((j^{\star N}_{n'} + \Lambda^N_{n'}y^{\star N}_{n'}) - 2(j^N_{n'} + \Lambda^N_{n'}y^N_{n'})\right) =$$
$$-nB^e_{nm} + (n+1)B^i_{nm} + \sum_{n',m',p,q}\frac{\varepsilon_N\chi^N_{pq}}{R}\Xi^{nm}_{n'm'pq}\left(-n'(n'-1)B^e_{n'm'} - (n'+1)(n'+2)B^i_{n'm'}\right)$$

$$B_{\theta,\mathrm{ext}}: \tag{S78}$$
$$\frac{1}{R}\left(a^N_{nm}j^{\star N}_n + b^N_{nm}y^{\star N}_n\right) + \sum_{n',m',p,q}\frac{\varepsilon_N\chi^N_{pq}}{R}\Xi^{\star\,nm}_{n'm'pq}\frac{a^N_{n'm'}}{R}\left((n'(n'+1) - k^2R^2)(j^N_{n'} + \Lambda^N_{n'}y^N_{n'}) - (j^{\star N}_{n'} + \Lambda^N_{n'}y^{\star N}_{n'})\right) =$$
$$-B^e_{nm} - B^i_{nm} + \sum_{n',m',p,q}\frac{\varepsilon_N\chi^N_{pq}}{R}\Xi^{\star\,nm}_{n'm'pq}\left(-(n'-1)B^e_{n'm'} + (n'+2)B^i_{n'm'}\right).$$

The new terms are all contained within the series in each equation. Because these terms are all multiplied by a factor ε_l/r_l, we can insert the spherically symmetric solutions for every term within the series, since we assume terms to second order in ε_l/r_l are negligible. Inserting expressions from Eqs. S36 and S50, we find that every single term within the series in the radial boundary conditions has a matching term on both sides, and so they all cancel. In the tangential equations, the only terms that survive are those that multiply an additional factor of k because it is different above and below the boundary. Thus, the boundary condition equations reduce to:

$$a^l_{nm}j^{l,l}_n + b^l_{nm}y^{l,l}_n = a^u_{nm}j^{u,l}_n + b^u_{nm}y^{u,l}_n \tag{S79}$$

$$a^l_{nm}j^{\star l,l}_n + b^l_{nm}y^{\star l,l}_n = a^u_{nm}j^{\star u,l}_n + b^u_{nm}y^{\star u,l}_n + \sum_{n',m',p,q}\frac{\varepsilon_l\chi^l_{pq}}{\bar{r}_l}\Xi^{\star\,nm}_{n'm'pq}\,\bar{a}^l_{n'm'}\left(j^{l,l}_{n'} + \Lambda^l_{n'}y^{l,l}_{n'}\right)\left(k_l^2r_l^2 - k_u^2r_l^2\right) \tag{S80}$$

$$\frac{n(n+1)}{R}\left(a^N_{nm}j^N_n + b^N_{nm}y^N_n\right) = -nB^e_{nm} + (n+1)B^i_{nm} \tag{S81}$$

$$\frac{1}{R}\left(a^N_{nm}j^{\star N}_n + b^N_{nm}y^{\star N}_n\right) = -B^e_{nm} - B^i_{nm} + \sum_{n',m',p,q}\frac{\varepsilon_N\chi^N_{pq}}{R}\Xi^{\star\,nm}_{n'm'pq}\frac{2n'+1}{n'+1}B^e_{n'm'}\frac{\xi^N_{n'} + \Lambda^N_{n'}\rho^N_{n'}}{\delta^N_{n'} + \Lambda^N_n\epsilon^N_{n'}}, \tag{S82}$$

with

$$\xi_{n'}^{N} \equiv -(kR)^2 j_n(kR) \tag{S83}$$
$$\rho_{n'}^{N} \equiv -(kR)^2 y_n(kR), \tag{S84}$$

and the bar over $\overline{a}_{n'm'}^{l}$ to indicate that it is identically the solution from the spherically symmetric case. The symmetric boundary condition equations give us

$$\overline{a}_{nm}^{\mathrm{i}} \equiv -\frac{2n+1}{n+1}\frac{RB_{nm}^{e}}{\delta_n^N + \Lambda_n^N \epsilon_n^N}\prod_{\mathrm{j=i+1}}^{N}\frac{j_n^{\mathrm{j,j-1}} + \Lambda_n^{\mathrm{j}} y_n^{\mathrm{j,j-1}}}{j_n^{\mathrm{j-1,j-1}} + \Lambda_n^{\mathrm{j-1}} y_n^{\mathrm{j-1,j-1}}}. \tag{S85}$$

With these expressions, and recalling that $b_{nm}^{1} = 0$ for all n and m, Eqs. S79–S82 are at last a solvable linear system of equations. Ultimately, for B_{nm}^{i} we obtain a solution

$$B_{nm}^{i} = \frac{n}{n+1}\mathcal{A}_n^e B_{nm}^e + n\sum_{\mathrm{i}=1}^{N}\mathcal{A}_n^{t,\mathrm{i}} K_n^{\mathrm{i}}\Delta_{nm}^{\mathrm{i}}, \tag{S86}$$

where we have defined

$$\mathcal{A}_n^{t,\mathrm{i}} \equiv \frac{j_n^{\mathrm{i,i}} + \Lambda_n^{\mathrm{i}} y_n^{\mathrm{i,i}}}{\delta_n^N + \Lambda_n^N \epsilon_n^N}, \tag{S87}$$

$$K_n^{\mathrm{i}} \equiv \prod_{\mathrm{j=i+1}}^{N}\frac{\alpha_n^{\mathrm{j,j}}}{\beta_n^{\mathrm{j,j-1}} + \Lambda_n^{\mathrm{j-1}}\gamma_n^{\mathrm{j,j-1}}}, \qquad K_n^N = 1, \tag{S88}$$

$$\Delta_{nm}^{\mathrm{i}} \equiv \begin{cases} \displaystyle\sum_{n',m',p,q}\frac{\varepsilon_{\mathrm{i}}\chi_{pq}^{\mathrm{i}}}{\overline{r}_{\mathrm{i}}}\Xi^{\star\,nm}_{n'm'pq}\frac{\overline{a}_{nm}^{\mathrm{i}}}{R}\left(j_{n'}^{\mathrm{i,i}} + \Lambda_{n'}^{\mathrm{i}} y_{n'}^{\mathrm{i,i}}\right)\left(k_{\mathrm{i}}^2\overline{r}_{\mathrm{i}}^2 - k_{\mathrm{i+1}}^2\overline{r}_{\mathrm{i}}^2\right) & \text{for i} < N, \\ \displaystyle\sum_{n',m',p,q}\frac{\varepsilon_N\chi_{pq}^N}{R}\Xi^{\star\,nm}_{n'm'pq}\frac{2n'+1}{n'+1}\mathcal{A}_{n'}^{\star}B_{n'm'}^{e} & \text{for i} = N, \end{cases} \tag{S89}$$

$$\mathcal{A}_{n'}^{\star} \equiv \frac{\xi_{n'}^N + \Lambda_{n'}^N\rho_{n'}^N}{\delta_{n'}^N + \Lambda_{n'}^N\epsilon_{n'}^N}. \tag{S90}$$

As with $\mathcal{A}_n^e$, the product $\mathcal{A}_n^{t,N}\mathcal{A}_{n'}^{\star}$ is asymptotic to $(1+0i)$ in the limit $|kR| \to \infty$ for all n and n'. Each of these complex amplitude quantities is labeled with a superscript to indicate their relationship to other relevant quantities: $\mathcal{A}^e$ multiplies the excitation field, $\mathcal{A}^t$ multiplies the tangential first-order term in the non-spherical expansion, and $\mathcal{A}^\star$ multiplies the mixing coefficients $\Xi^\star$. The radial first-order term that would multiply a quantity $\mathcal{A}^r$ analogous to $\mathcal{A}^t$ is identically zero, so it does not appear. Eq. S86 is our final result, and may be used to evaluate the induced magnetic field for any arbitrary layered conducting body, so long as the near-spherical approximation holds for each boundary.

S2. Table of interior structure parameters

In Table S1, we list the chosen bulk properties for the four satellites investigated here. The base *PlanetProfile* models assume spherical symmetry. The entire set of assumed parameters can be found in the Matlab files for the individual models, which can also be used to reproduce the models themselves. Those files are found in the Supplemental Material.

S3. Gravitational deformation in satellites; application to Europa

Tidal forces applied by gravity from the parent planet and centrifugal acceleration from spin rotation will deform satellites, primarily in the $p = 2$ spherical harmonic shapes (Rambaux and Castillo-Rogez, 2013). Anderson et al.

Body	T_s (K)	R (km)	M (kg)	C/MR^2	Reference
Europa	110	1561.0	4.7991×10^{22}	0.346 ± 0.005	Anderson et al. (1998)
Callisto	110	2410.3	1.4819×10^{23}	0.3549 ± 0.0042	Anderson et al. (2001)
Miranda	60	235.8	6.4×10^{19}	0.346	Hussmann et al. (2006)
Triton	38	1353.4	2.14×10^{22}	0.315	Hussmann et al. (2006)

Table S1
Model parameters used to determine interior conductivity profiles using *PlanetProfile*. T_s: surface temperature; R: radius of body surface; M: total body mass; C/MR^2: axial coefficient of moment of inertia. Moments of inertia for Miranda and Triton are assumed for consistency with prior models, as no measurements are available. For Triton the value was increased by 0.005 for consistency with the model study of Cochrane et al. (2021).

(1998) used Doppler shifts available from precise radio tracking of *Galileo* by the Deep Space Network to infer the $p = 2$ gravity coefficients for Europa. These authors favored best-fit values of $C_{20} = -435.5 \times 10^{-6}$, $C_{22} = 131.0 \times 10^{-6}$ from their analysis. Assuming the body is in hydrostatic equilibrium, these authors also found the axial moment of inertia for Europa to be approximately $C/MR^2 = 0.346$.

Under the assumption of hydrostatic equilibrium, the Radau–Darwin approximation allows us to relate the axial moment of inertia to the secular (non-time-varying) Love number k_f (Rambaux and Castillo-Rogez, 2013):

$$\frac{C}{MR^2} = \frac{2}{3}\left[1 - \frac{2}{5}\sqrt{\frac{4-k_f}{1+k_f}}\right]. \tag{S91}$$

The fluid Love number $h_f = k_f + 1$. This quantity relates the gravitational tides to the geometric deformation of the body by (Rambaux and Castillo-Rogez, 2013; Hemingway and Mittal, 2019)

$$r_{\mathrm{surf}}(\theta,\phi) = h_f \frac{V(\theta,\phi)}{g_{\mathrm{surf}}}, \tag{S92}$$

where r_{surf} is the shape of the body surface, V is the gravitational potential, and $g_{\mathrm{surf}} = GM/R^2$ is the mean gravitational acceleration at the surface. At the surface of the body, the gravitational moments V_{pq} are proportional to the coefficients C_{pq} with a proportionality factor of GM/R (Rambaux and Castillo-Rogez, 2013). Inserting gravitational terms into Eq. S92, we arrive at the simple conversion

$$H_{pq} = h_f C_{pq} R, \tag{S93}$$

where H are 4π-normalized spherical harmonic coefficients for the body surface shape—analogous to χ^N_{pq} (Eq. S59), but in a different normalization that matches the one in which the gravity coefficients are given. Eq. S93 will allow us to find the shape of the body surface as perturbed by tidal forces.

We can now determine the values to apply to Europa. Solving Eq. S91 for k_f and replacing with h_f, we obtain

$$h_f = 1 + \frac{4-u}{1+u}, \quad u \equiv \left[\frac{5}{2}\left(1 - \frac{3}{2}\frac{C}{MR^2}\right)\right]^2, \tag{S94}$$

with $h_f = 2.044$ for Europa from $C/MR^2 = 0.346$ as determined by Anderson et al. (1998). Combining these results with Eq. S93 and the C_{20}, C_{22} gravity coefficients from Anderson et al. (1998), we obtain 4π-normalized shape coefficients of

$$H_{20} = -1.390\,\mathrm{km}, \tag{S95}$$
$$H_{22} = 0.418\,\mathrm{km}. \tag{S96}$$

These values describe the equilibrium shape of the icy surface of Europa as perturbed by tides. In order to preserve the ice shell thickness estimates we model after Tobie et al. (2003), we apply the same gravitational shape perturbation to the surface and ocean layers, without scaling.

Although Enceladus also has a triaxial ellipsoid shape perturbed by gravity (Iess et al., 2014), we choose not to apply this analysis to our Enceladus-like interior for Miranda because gravity data are not yet available for Miranda. Thus, we limit our Miranda models to the asymmetry in the ice–ocean boundary.

S4. Supplemental figures for example applications

In Figures S1–S6, we include a full detailing of analysis products from example applications of our model to several ocean worlds in the solar system (described in Section 3). Blue–white contour maps show the asymmetry models applied for all example cases studied: asymmetric ice–ocean boundaries for Europa and Miranda and asymmetric ionospheres for Triton and Callisto. Heat maps show the magnitude of the induced magnetic field predicted for the asymmetric models. Some maps are repeated from the main text for completeness. Symmetric analog models have the same layer structure as the asymmetric models, and are evaluated using the standard recursion method of Srivastava (1966). Please refer to the main text (Section 3) for further details regarding the reasoning behind each model.

Red–blue and other colormaps show the difference in induced magnetic field between our model predictions for the asymmetric cases as compared to spherically symmetric analogs, for all magnetic field vector components and the magnitude. Vector components are in IAU coordinates, such that at 0° latitude, 0° longitude, the parent planet is directly overhead; this is a right-handed coordinate system, with east longitudes positive. This coordinate system is rotated approximately 90° from the $\phi\Omega$ ("Phi-O," *e.g.* E-Phi-O for Europa) coordinates sometimes used in analysis of spacecraft data. All induced fields are evaluated at the J2000 reference epoch, 12:00 pm Jan 1, 2000 TDB and consider only the synodic period. Animations of the differences in magnitude and the B_x component throughout a synodic period are included for each model as Supplemental Material.

S4.1. Europa — effect size with distance

It may be advantageous for future investigations to pursue modeling efforts that neglect asymmetry, or at least limit studied cases to only include known shapes (*i.e.* tidal deformation). To facilitate these efforts, we have evaluated the difference in the induced magnetic field resulting from asymmetry as a function of distance from Europa. Figure S7 shows the difference in induced field caused by asymmetry from our Europa Seawater model (Figure S1) from the surface upward to 2000 km altitude. We selected a time (0.7 hr after J2000) and surface point (see Figure S1c) at which the effect size is approximately maximized, with a difference at the surface of about 2.2 nT. The difference resulting from asymmetry drops to 0.2 nT at about 1 R_E in altitude, around 1500 km.

It may also be noted that the effect of asymmetry on the induced field can become small at key points in time during the considered excitation period. In Figure S8, we have extracted a single frame from our animation of the B_x difference for the Europa Seawater model available in the Supplemental Material. At the time shown—3.4 hr past J2000—only the 0 nT contour is plotted, meaning the difference is everywhere less than 0.6 nT. This condition lasts for about 4% of the synodic period, or about 30 minutes. If a spacecraft reaches its closest approach near this time, at a distance at or above 25 km, the difference contributed by the asymmetric ocean will be small. This implies that there may be some conditions (including particular asymmetry models) under which the induced field measured by the spacecraft is essentially the same as that predicted by a spherically symmetric model.

S5. Direct expressions for $3j$-symbols and harmonic product coefficients

Products of spherical harmonics are often expressed in terms of the Wigner $3j$-symbols. The general expression for the $3j$-symbols is presented by several authors (*e.g.* Brink and Satchler, 1968; Edmonds, 1996):

$$\begin{pmatrix} j_1 & j_2 & J \\ m_1 & m_2 & M \end{pmatrix} = (-1)^{j_1-j_2-M} \sqrt{\frac{(j_1+j_2-J)!(j_1-j_2+J)!(-j_1+j_2+J)!}{(j_1+j_2+J+1)!}} \quad \times$$

$$\sqrt{(j_1+m_1)!(j_1-m_1)!(j_2+m_2)!(j_2-m_2)!(J+M)!(J-M)!} \quad \times \tag{S97}$$

$$\sum_\kappa \left[(-1)^\kappa \Big(\kappa!(j_1+j_2-J-\kappa)!(j_1-m_1-\kappa)!(j_2+m_2-\kappa)!(J-j_2+m_1+\kappa)!(J-j_1-m_2+\kappa)! \Big)^{-1} \right]$$

$$\text{for} \quad |j_1-j_2| \le J \le j_1+j_2 \quad \text{and} \quad M = -(m_1+m_2),$$

$$\begin{pmatrix} j_1 & j_2 & J \\ m_1 & m_2 & M \end{pmatrix} = 0 \quad \text{otherwise.}$$

The sum in Eq. S97 is over each integer value of κ for which *all* factorials in the sum are nonnegative. For many of the low-degree combinations of spherical harmonics with which we are concerned, the series has only a single term with $\kappa = 0$.

When $m_1 = m_2 = M = 0$, the $3j$-symbols are non-zero only if $j_1 + j_2 + J$ is even (Brink and Satchler, 1968). Another way to express this is that J takes values of $j_1 + j_2,\ j_1 + j_2 - 2,\ j_1 + j_2 - 4,\ \dots\ |j_1 - j_2|$. Eq. S97 simplifies under many conditions. The results of products of spherical harmonics are always proportional to $\begin{pmatrix} j_1 & j_2 & J \\ 0 & 0 & 0 \end{pmatrix}$. In this case, Eq. S97 becomes (Edmonds, 1996)

$$\begin{pmatrix} j_1 & j_2 & J \\ 0 & 0 & 0 \end{pmatrix} = (-1)^{(j_1+j_2+J)/2} \sqrt{\frac{(j_1+j_2-J)!(j_1-j_2+J)!(-j_1+j_2+J)!}{(j_1+j_2+J+1)!}} \times \frac{\left(\frac{1}{2}(j_1+j_2+J)\right)!}{\left(\frac{1}{2}(j_1+j_2-J)\right)!\left(\frac{1}{2}(j_1-j_2+J)\right)!\left(\frac{1}{2}(-j_1+j_2+J)\right)!} \tag{S98}$$

Using Eqs. S97 and S98, we may obtain explicit expressions for the products $Y_{nm}Y_{pq}$ and $Y^\star_{nm}Y_{pq}$. The selection rules for both mixing coefficients are similar, but not identical. For $\Xi^{n'm'}_{nmpq}$, the conditions are

$$|n-p| \le n' \le n+p, \quad n+p+n' \text{ is even}, \quad \text{and} \quad m' = m+q. \tag{S99}$$

For $\Xi^{\star\, n'm'}_{nmpq}$, the conditions are instead

$$0 < n' \le n+p, \quad n+p+n' \text{ is even}, \quad \text{and} \quad m' = m+q. \tag{S100}$$

For $\Xi^{\star\, n'm'}_{nmpq}$, the triangular condition is modified because it relates products involving $Y^\star_{nm}$, which are linear combinations of $Y_{n+1,m}/\sin\theta$ and $Y_{n-1,m}/\sin\theta$, linking each (n,m) to both $(n+2,m)$ and $(n-2,m)$. The net result is that either $n' = 1$ or 2 will have non-zero mixing coefficients for all combinations of n and p, and even large p can still impact induced moments of degree 1 (the dipole moments).

If the selection rules are satisfied, the following expressions apply. Otherwise, the coefficient is zero. In the radial boundary conditions, the coefficients are

$$\begin{aligned}
Y_{nm}Y_{pq} &= \sum_{n'} \Xi^{n'm'}_{nmpq} Y_{n'm'} \\
\Xi^{n'm'}_{nmpq} &= (-1)^\nu \sqrt{\frac{(2n+1)(2p+1)(2n'+1)}{4\pi}} \frac{(2\nu)!}{\nu!} \frac{(2n-2\nu)!}{(n-\nu)!} \frac{(2p-2\nu)!}{(p-\nu)!} \frac{(n'+\nu)!}{(2n'+1+2\nu)!} \times \\
&\quad \sqrt{(n+m)!(n-m)!(p+q)!(p-q)!(n'+m')!(n'-m')!} \times \\
&\quad \sum_{\kappa=\kappa^-}^{\kappa^+} \left[(-1)^\kappa \Big(\kappa!(2\nu-\kappa)!(n-m-\kappa)!(n+m-(2\nu-\kappa))!(p+q-\kappa)!(p-q-(2\nu-\kappa))!\Big)^{-1}\right], \\
&\text{with } \nu \equiv \frac{1}{2}(n+p-n').
\end{aligned} \tag{S101}$$

The sum over κ is again limited to those integer values of κ for which all factorials have nonnegative arguments. This limits possible values of κ to lie between 0 and 2ν, but does not necessarily include those values. For example, in the case of $q = p$ with $\nu = 1$, $\kappa = 0$ yields a negative value for $p - q - (2\nu - \kappa)$, the final factorial in the series expression. This occurs for $n, m = 1, -1$, $p, q = 2, 2$, $n', m' = 1, 1$, a case of interest for application of our methods because it relates $p = 2$ boundary harmonics to the induced dipole moment from a uniform excitation field. This condition dictates that the minimum value for κ is the greatest value among $[0,\ 2\nu - (n+m),\ 2\nu - (p-q)]$ and the maximum value for κ is the least value among $[2\nu,\ n-m,\ p+q]$. In summary:

$$\begin{aligned}
\kappa^- &= \max(0,\ 2\nu - (n+m),\ 2\nu - (p-q)) \\
\kappa^+ &= \min(2\nu,\ n-m,\ p+q).
\end{aligned} \tag{S102}$$

In the tangential boundary conditions, the products $Y^\star_{nm}Y_{pq}$ proportional to a desired $Y^\star_{n'm'}$ may be replaced by $\Xi^{\star\,n'm'}_{nmpq}$:

$$\Xi^{\star\,n'm'}_{nmpq} = \frac{\Xi^w_{n'} + \sum_{g=1}^{g^-_{\max}} \Xi^w_{n'-2g} \prod_{\kappa=0}^{g-1} F^-_{n',2\kappa} + \sum_{g=1}^{g^+_{\max}} \Xi^w_{n'+2g} \prod_{\kappa=0}^{g-1} F^+_{n',2\kappa}}{\left(w^-_{n'}\right)^2 + \left(w^+_{n'}\right)^2 - w^-_{n'}w^+_{n'-2}F^-_{n',0} - w^+_{n'}w^-_{n'+2}F^+_{n',0}}, \tag{S103}$$

$$g^-_{\max} \equiv \begin{cases} \mathrm{floor}\left(\frac{1}{2}\left(n' - |m'|\right)\right) & \text{if } n' \geq 3 \\ 0 & \text{otherwise} \end{cases}, \quad g^+_{\max} \equiv \mathrm{floor}\left(\frac{1}{2}\left(n_{\max} + p_{\max} - n'\right)\right), \tag{S104}$$

$$F^\pm_{n,2\kappa} \equiv \frac{w^\pm_{n\pm2\kappa}w^\mp_{n\pm(2\kappa+2)}}{\left(w^-_{n\pm(2\kappa+2)}\right)^2 + \left(w^+_{n\pm(2\kappa+2)}\right)^2 - w^\pm_{n\pm(2\kappa+2)}w^\mp_{n\pm(2\kappa+4)}F^\pm_{n,2\kappa+2}}, \tag{S105}$$

$$\Xi^w_{n'} \equiv w^-_{nm}w^-_{n'm'}\Xi^{n'-1\,m'}_{n-1\,mpq} + w^+_{nm}w^+_{n'm'}\Xi^{n'+1\,m'}_{n+1\,mpq} - w^-_{nm}w^+_{n'm'}\Xi^{n'+1\,m'}_{n-1\,mpq} - w^+_{nm}w^-_{n'm'}\Xi^{n'-1\,m'}_{n+1\,mpq}, \tag{S106}$$

$$w^-_{nm} = (n+1)\sqrt{\frac{n^2 - m^2}{(2n-1)(2n+1)}}, \quad w^+_{nm} = n\sqrt{\frac{(n+1)^2 - m^2}{(2n+1)(2n+3)}}, \tag{S107}$$

where $\kappa = 0, 1, 2, \ldots$, $\mathrm{floor}(a)$ rounds a down to the nearest integer, and a subscript m is implied on all w in all equations where it is omitted, corresponding to the subscript n. The recursion in $F^+_{n,2\kappa}$ continues until 2κ exceeds $n_{\max} + p_{\max} - n$, and the recursion in $F^-_{n,2\kappa}$ continues until 2κ exceeds $n - |m| - 2$. When 2κ exceeds these respective values, $F^\pm_{n,2\kappa} = 0$. $F^-_{n,2\kappa}$ is also zero for $n < 3$. The sums in the numerator of Eq. S103 result from isolating the desired terms of degree n' and order m'; the denominator results from solving the isolated term for $\Xi^{\star\,n'm'}_{nmpq}$.

These bounding values result from non-orthogonality of the $Y^\star_{nm}$. For a term of degree n'' below the considered value n', non-orthogonality is possible only for $n'' = n' - 2$, $n' - 4$, etc., but $Y^\star_{n''m''}$ is defined and non-zero only for $n'' > 0$ and $n'' \geq m''$. This constraint limits the non-zero values of $F^-_{n',0}$ to $n' \geq 3$ and $n' \geq m' + 2$. The n'' above the considered n' that yield non-orthogonal terms are similarly limited by the selection rules for $\Xi^{n'm'}_{nmpq}$, *i.e.* n'' cannot exceed $n_{\max} + p_{\max}$ because no combination of $Y^\star_{nm}Y_{pq} \to \Xi^{\star\,n'm'}_{nmpq}Y^\star_{n'm'}$ results in $n' > n_{\max} + p_{\max}$ and we have assumed the series in n' may be truncated at $n' = n_{\max} + p_{\max}$.

S6. Sharp transitions in layer conductivities

As a matter of practical consideration, here we highlight important challenges for application of the layer method (and thus our results) and strategies for their mitigation. The content of this section has also proven useful for validating the functional dependence of the quantities we derive in our solutions. In this section, we assume spherical symmetry.

The Bessel functions j_n and y_n always contain complex exponentials with complex arguments kr. For large $|kr|$, the functions are all asymptotic to a growing exponential divided by kr; when kr is close to zero, they are asymptotic to powers of kr (Marion and Heald, 1980). On planetary scales, kr often takes extreme values in various layers. For example, in metallic cores, the conductivity σ may be over 10^6 S/m (Khurana et al., 2002), providing a strong response for all periods of excitation and for any value of r. $|kr|$ can be large for even moderately conducting oceans on large scales—$|kr| \sim 100$ for a spherical Earth-size ocean for a 1-day period of oscillation. In contrast, in insulating materials such as ice and rock, conductivity values can be extremely low: $10^{-12} \lesssim \sigma \lesssim 10^{-2}$, depending on hydration state, porosity, and presence of contaminants (Glover, 2015). Additionally, for all materials, conductivity is pressure- and temperature-dependent. Because $|kr|$ can span many orders of magnitude across boundaries, especially at an ice–ocean interface or a core–mantle boundary, application of the recursion relations in Section S1.3.1 often results in differences of very large terms that are very close together in value, requiring great numerical precision in computation to reliably evaluate. With some realistic planetary values, numerical overflow or underflow are assured even for specialized, high-precision libraries such as mpmath[2] in Python and MPFUN[3] in Fortran.

[2] http://mpmath.org/

[3] MPFUN is available for Unix-based systems on author D. Bailey's personal website: https://www.davidhbailey.com/dhbsoftware/

In handling these challenges, we have found it essential to account for sharp conductivity boundaries by using approximations appropriate to the type of boundary transition. Under limiting conditions, the Bessel functions take the following asymptotic forms, derived from Marion and Heald (1980):

$$\begin{aligned} j_n(kr) &\approx \frac{(-i)^n e^{ikr}-(i)^n e^{-ikr}}{2ikr}, \quad y_n(kr) \approx \frac{-(-i)^n e^{ikr}-(i)^n e^{-ikr}}{2kr}, \\ j_n^\star(kr) &\approx \frac{(-i)^n e^{ikr}+(i)^n e^{-ikr}}{2}, \quad y_n^\star(kr) \approx \frac{(-i)^n e^{ikr}-(i)^n e^{-ikr}}{2i} \end{aligned} \qquad \text{for } |kr| \gg n, \tag{S108}$$

$$\begin{aligned} j_n(kr) &\approx \frac{(kr)^n}{(2n+1)!!}, \quad y_n(kr) \approx -\frac{(2n-1)!!}{(kr)^{n+1}}, \\ j_n^\star(kr) &\approx (n+1)\frac{(kr)^n}{(2n+1)!!}, \quad y_n^\star(kr) \approx n\frac{(2n-1)!!}{(kr)^{n+1}} \end{aligned} \qquad \text{for } |kr| \ll n. \tag{S109}$$

These expressions may then be used to identify replacement rules for the Bessel functions as they appear in Eq. S38 for extreme layers:

$$\begin{aligned} j_n^\star(kr) &\to -kr\, y_n(kr), \quad y_n^\star(kr) \to kr\, j_n(kr), \\ |j_n^\star(kr)| &\gg |j_n(kr)|, \quad |y_n^\star(kr)| \gg |y_n(kr)| \end{aligned} \qquad \text{for } |kr| \gg n, \tag{S110}$$

$$\begin{aligned} j_n^\star(kr) &\to (n+1)\, j_n(kr), \quad y_n^\star(kr) \to -n\, y_n(kr), \\ |y_n(kr)| &\gg |j_n(kr)|, \quad |y_n^\star(kr)| \gg |j_n^\star(kr)| \end{aligned} \qquad \text{for } |kr| \ll n. \tag{S111}$$

Using this information, we can reduce Eqs. S38 and S56 to account for extreme transitions. For the innermost boundary, Eq. S38 becomes

$$\Lambda_{nm}^2 \to -\frac{j_n^{2,1} + \frac{j_n^{\star 2,1}}{ik_1 r_1}}{y_n^{2,1} + \frac{y_n^{\star 2,1}}{ik_1 r_1}} \qquad \text{for } |k_1 r_1| \gg n, \tag{S112}$$

$$\Lambda_{nm}^2 \to -\frac{j_{n+1}^{2,1}}{y_{n+1}^{2,1}} \qquad \text{for } |k_1 r_1| \ll n. \tag{S113}$$

Note that the superscripts in the expressions above are all indices, not exponents—these relate the quantity Λ for the second-innermost layer to k_1, k_2, and r_1 when the innermost layer is a very strong or very poor conductor.

For intermediate layers, when $|kr|$ is large, if we keep only first-order terms in $1/kr$ we obtain the same result as Eq. S112. Consequently, if $|kr|$ is large enough to cause numerical overflows, currents flowing in this layer entirely screen every layer beneath it from oscillations in the magnetic field. The behavior is more complicated for an interstitial nonconducting layer, however.

For a jth (middle) layer with wavenumber k_j, lower bounding radius $r_l = r_{\mathrm{j}-1}$, upper bounding radius $r_u = r_\mathrm{j}$, and wavenumbers $k_l = k_{\mathrm{j}-1}$, $k_u = k_{\mathrm{j}+1}$ pertaining to the lower and upper layers respectively, Λ_{nm}^u for the upper layer takes the form

$$\Lambda_{nm}^u \to -\frac{j_n^{u,u} + \frac{j_n^{\star u,u}}{ik_\mathrm{j} r_\mathrm{j}}}{y_n^{u,u} + \frac{y_n^{\star u,u}}{ik_\mathrm{j} r_\mathrm{j}}} \qquad \text{for } |k_\mathrm{j} r_\mathrm{j}| \gg n, \tag{S114}$$

$$\Lambda_{nm}^u \to \frac{\beta_n^U + \mathcal{A}_{nm}^L \delta_n^U \left(\frac{r_l}{r_u}\right)^{2n+1}}{\gamma_n^U + \mathcal{A}_{nm}^L \epsilon_n^U \left(\frac{r_l}{r_u}\right)^{2n+1}} = -\frac{j_{n+1}(k_u r_u) - \mathcal{A}_{nm}^L j_{n-1}(k_u r_u)\left(\frac{r_l}{r_u}\right)^{2n+1}}{y_{n+1}(k_u r_u) - \mathcal{A}_{nm}^L y_{n-1}(k_u r_u)\left(\frac{r_l}{r_u}\right)^{2n+1}} \qquad \text{for } |k_\mathrm{j} r_\mathrm{j}| \ll n. \tag{S115}$$

In Eq. S115 we have defined the following quantities based on their similarity to those that appear in Eqs. S51–S54 and S39:

$$\beta_n^U = j_n^\star(k_u r_u) - (n+1) j_n(k_u r_u) \qquad = -k_u r_u j_{n+1}(k_u r_u) \tag{S116}$$

$$\gamma_n^U = y_n^\star(k_u r_u) - (n+1) y_n(k_u r_u) \qquad = -k_u r_u y_{n+1}(k_u r_u) \tag{S117}$$

$$\delta_n^U = j_n^\star(k_u r_u) + n j_n(k_u r_u) \qquad = k_u r_u j_{n-1}(k_u r_u) \tag{S118}$$

$$\epsilon_n^U = y_n^\star(k_u r_u) + n y_n(k_u r_u) \qquad = k_u r_u y_{n-1}(k_u r_u) \tag{S119}$$

$$\mathcal{A}_{nm}^L = \frac{\beta_n^l + \Lambda_{nm}^l \gamma_n^l}{\delta_n^l + \Lambda_{nm}^l \epsilon_n^l} \qquad = -\frac{j_{n+1}(k_l r_l) + \Lambda_{nm}^l y_{n+1}(k_l r_l)}{j_{n-1}(k_l r_l) + \Lambda_{nm}^l y_{n-1}(k_l r_l)}, \tag{S120}$$

where β_n^l–ϵ_n^l in Eq. S120 are defined as in Eqs. S51–S54—the lower boundary acts as an "outer" boundary, as Laplace's equation applies in the space where $k \to 0$. Eq. S115 is the only layer recursion relation that contains more than one radius value, because it propagates from the lower boundary to the upper across the "gap" nonconducting layer. Finally, at the outermost boundary:

$$\mathcal{A}_n^e \to 1 \qquad |kR| \gg n, \tag{S121}$$

$$\mathcal{A}_n^e \to \left(\frac{r_{N-1}}{R}\right)^{2n+1} \frac{\beta_n^{N-1} + \Lambda_n^{N-1}\gamma_n^{N-1}}{\delta_n^{N-1} + \Lambda_n^{N-1}\epsilon_n^{N-1}} \qquad |kR| \ll n. \tag{S122}$$

For a highly conducting outermost layer, all interior layers are screened and the entire body acts as a perfect conductor. For an insulating outer layer, Eq. S122 tells us the net effect is that the outer boundary moves one layer down and the final value for $\mathcal{A}_n^e$ is scaled down as if the outer layer were not present.

Eqs. S112–S122 have been constructed such that numerical overflow is avoided in computation. In this formulation, when underflow occurs, a valid approximate result is still successfully computed. These expressions are physically valid when the skin depth $s_\mathrm{j} = 1/\mathrm{Im}(k_\mathrm{j})$ is much less or much greater than the layer thickness $D_\mathrm{j} = r_\mathrm{j} - r_{\mathrm{j}-1}$ (Styczinski and Harnett, 2021).

Additional references

Anderson J.D., Jacobson R.A., McElrath T.P., Moore W.B., Schubert G., Thomas P.C. Shape, mean radius, gravity field, and interior structure of Callisto Icarus, 153 (1) (2001), pp. 157-161.

Arfken G., Weber H., Harris F. Mathematical Methods For Physicists (seventh ed.), Academic Press (2012).

Backus G. Poloidal and toroidal fields in geomagnetic field modeling Rev. Geophys., 24 (1) (1986), pp. 75-109.

Backus G., George B., Parker R.L., Parker R., Constable C. Foundations of Geomagnetism Cambridge University Press (1996).

Brink D.M., Satchler G.R. Angular Momentum (second ed.), Clarendon Press (1968).

Dennery P., Krzywicki A. Mathematics for Physicists Dover (2012).

Edmonds A.R. Angular Momentum in Quantum Mechanics Princeton University Press (1996).

Rambaux N., Castillo-Rogez J. Tides on Satellites of Giant Planets Souchay J., Mathis S., Tokieda T. (Eds.), Tides in Astronomy and Astrophysics, Springer-Verlag Berlin Heidelberg (2013), pp. 167-200, doi: 10.1007/978-3-642-32961-6_5.

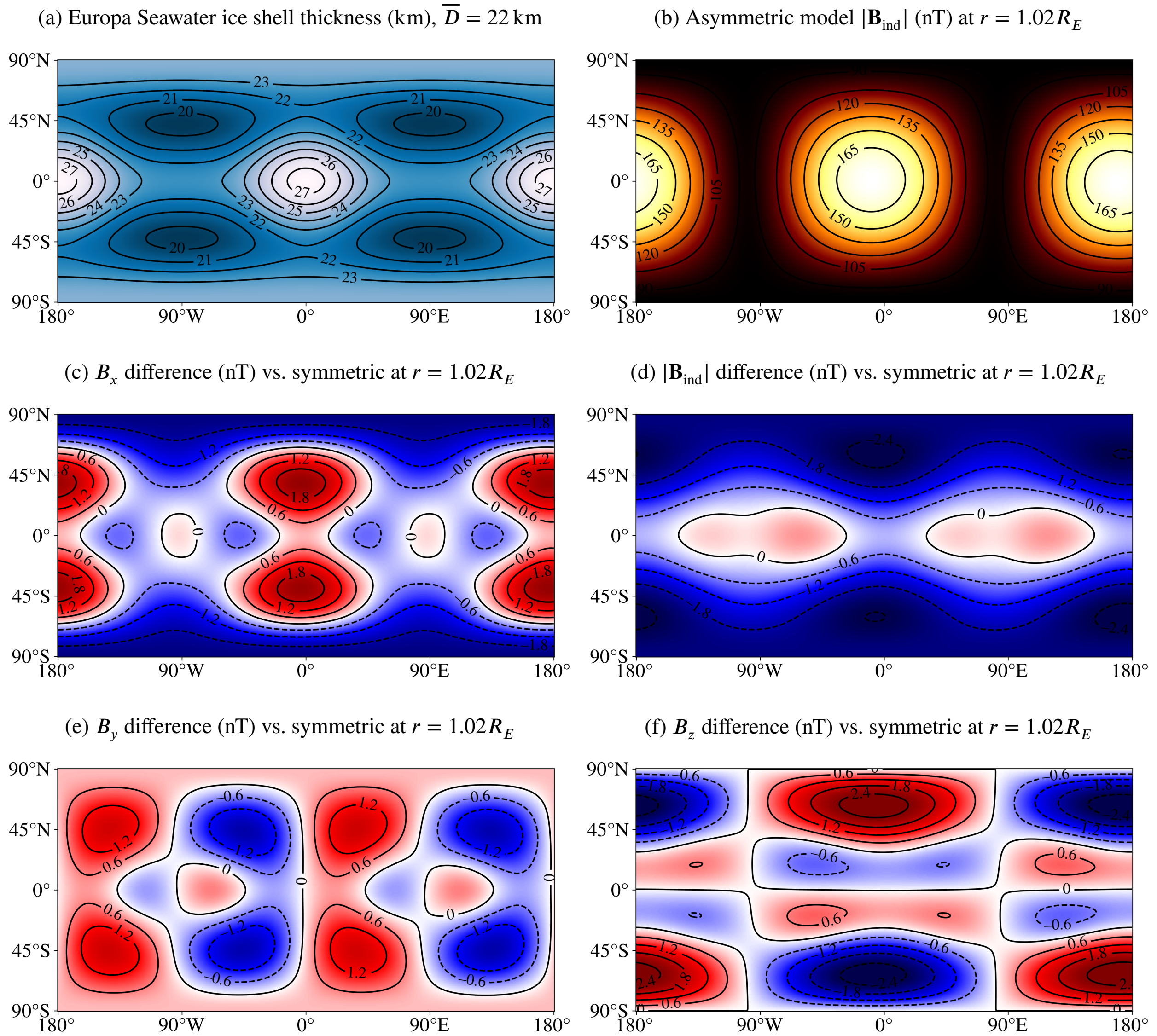

Figure S1: Europa model with an asymmetric ice–ocean boundary approximating the results of Tobie et al. (2003), whose analysis was based on modeling tidal heating and thermodynamics, and static gravity inferred by Anderson et al. (1998). Compare Figure S1a to Figure 12a of Tobie et al. (2003). Average ice shell thickness is 22.5 km. In this model, a Seawater composition is assumed for the ocean; conduction in the ionosphere is ignored. Electrical conductivities are calculated using the *PlanetProfile* geophysical modeling framework (Vance et al., 2021). Magnetic fields are evaluated at the J2000 epoch and at 25 km altitude, as the upcoming *Europa Clipper* mission plans several flybys of 25 km or less at closest approach. Only the synodic period is modeled here for simplicity. The difference in the magnetic field resulting from asymmetry is over 2 nT in some locations, and is likely to have a measurable influence on *Europa Clipper* investigations using data from these near flybys. The differences in induced field are not static but move and oscillate throughout the synodic period. Animations for the difference in x component and magnitude are included as Supplemental Material.

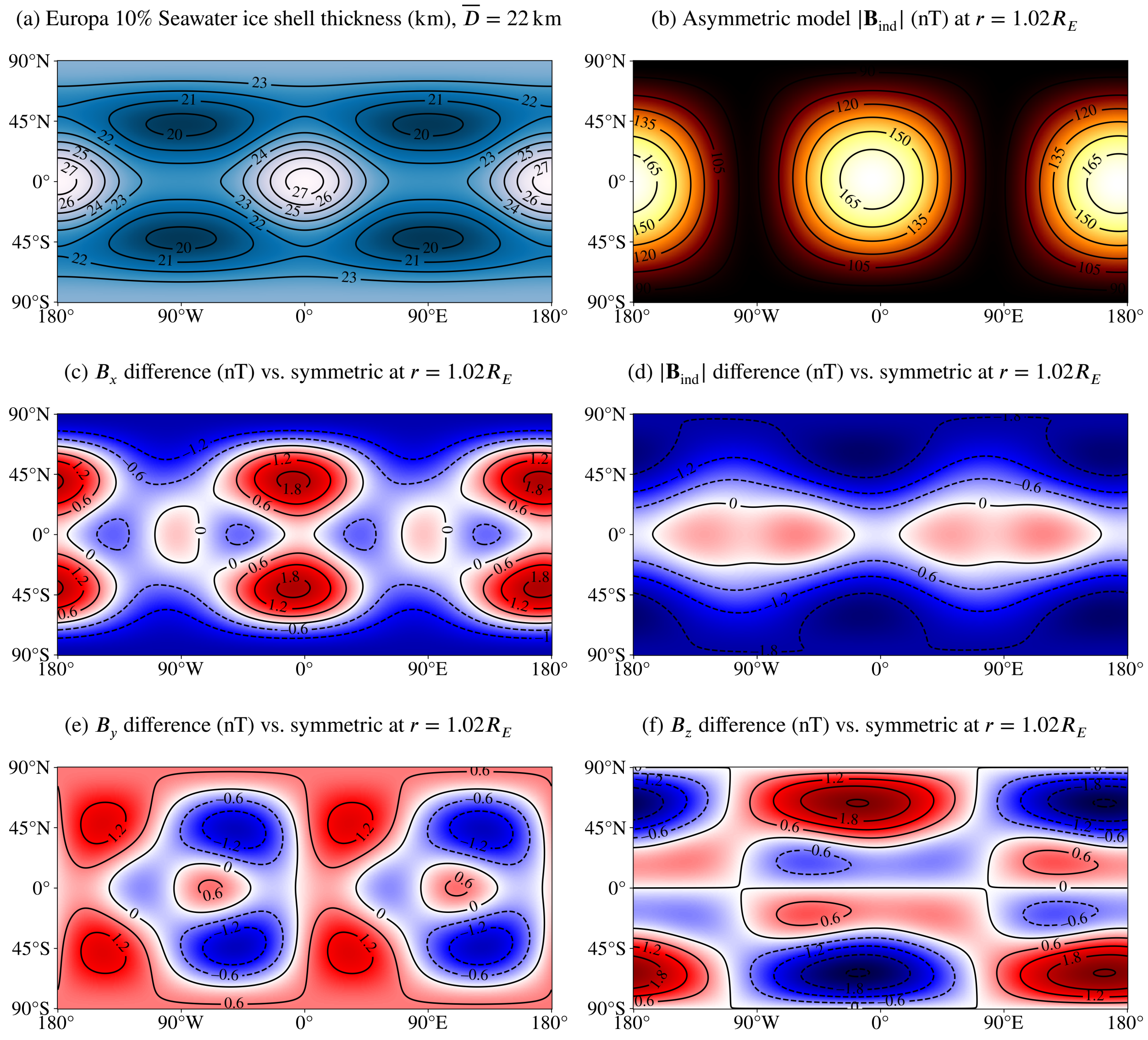

Figure S2: Europa model very similar to that in Figure S1 but with an ocean composition 1/10 the salinity of Seawater. Magnetic fields are again evaluated at the J2000 epoch and at 25 km altitude. Comparison to Figure S1 shows smaller differences and a slight phase difference. However, the differences in the magnetic field resulting from asymmetry are still well over 1 nT in some places even in this lower-salinity case, a consequence of Europa's relatively large size. The colormaps and contours for the difference plots are fixed to match those of Figure S1. Animations for the difference in x component and magnitude as they vary throughout the synodic period are included as Supplemental Material.

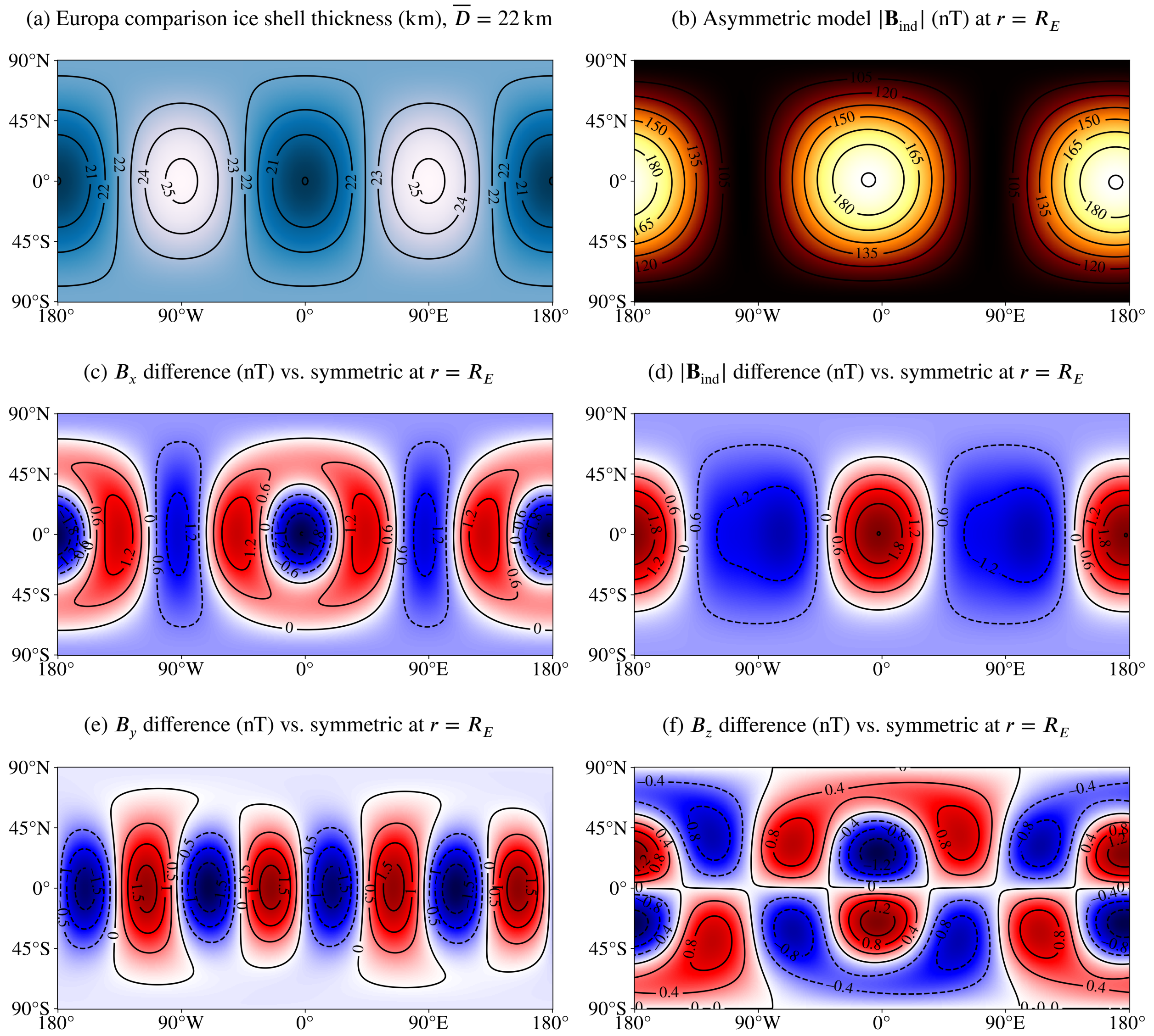

Figure S3: Simplified Europa model also based on the results of Tobie et al. (2003), but with only a single real harmonic represented in the boundary shape and with very high ocean conductivity. The asymmetry model is identical to that considered in our previous work (Styczinski and Harnett, 2021); in this case, the conductivity is very high ($2,750\,\mathrm{S/m}$) so that the result can be compared to the high-conductivity limit previously studied. Conduction in the ionosphere is ignored. Magnetic fields are evaluated at the J2000 epoch on the surface of Europa. For simplicity, only the synodic period is modeled here. As can be seen in the time series for B_x at the sub-jovian point plotted in Figure 5 (main text), the more rigorous model derived in this work predicts a larger difference compared to the spherically symmetric case, several times larger than our previous estimates. Animations for the difference in x component and magnitude as they vary throughout the synodic period are included as Supplemental Material.

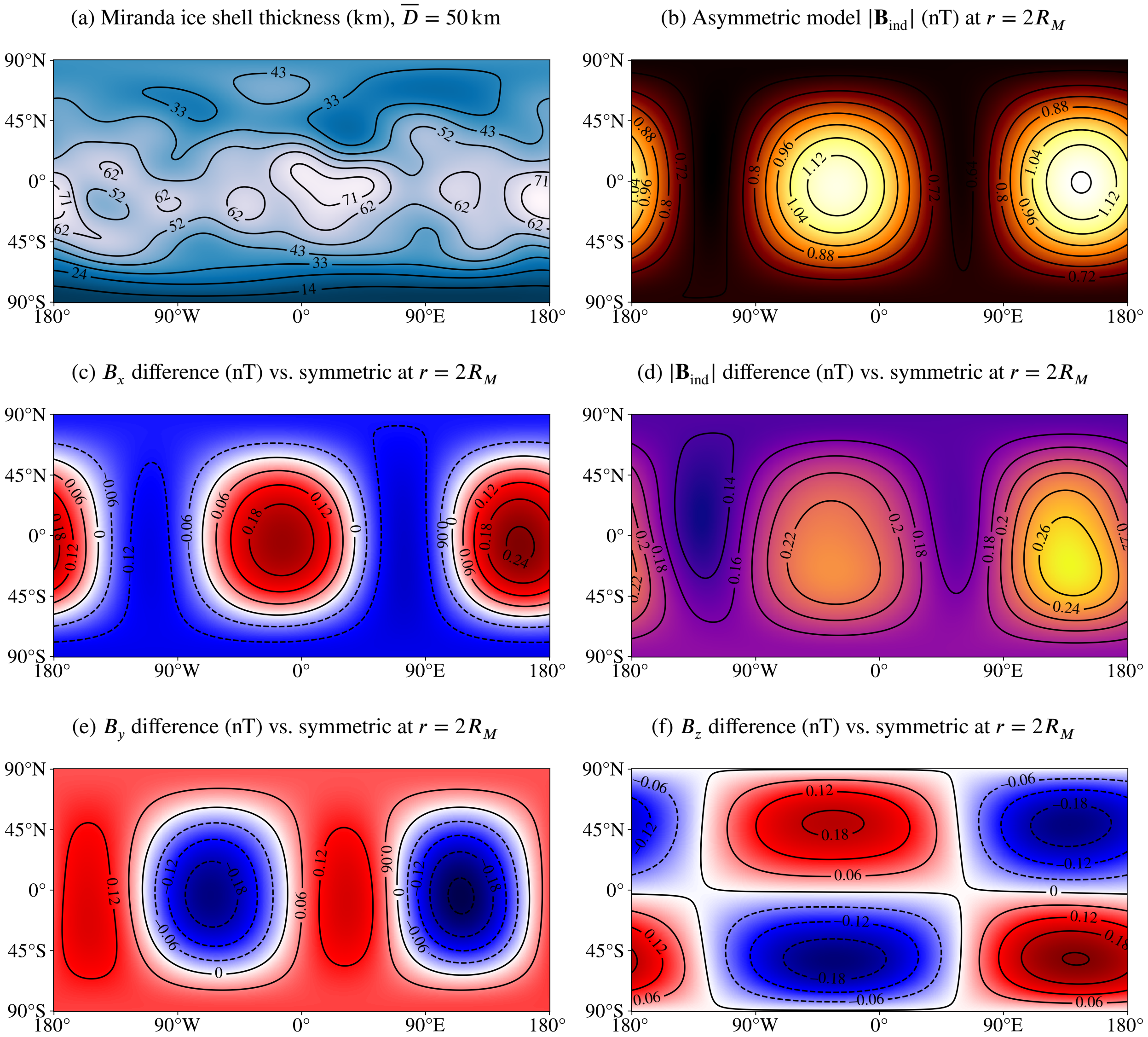

Figure S4: Miranda model with an ice–ocean boundary shape based on the asymmetric Enceladus ice shell inferred by Hemingway and Mittal (2019) from isostatic compensation and gravity measurements. The asymmetry in the ice shell has been scaled up from the 21 km average thickness model of Enceladus to the 50 km average thickness ice shell we suppose for Miranda, to serve as an upper limit of expected asymmetry in demonstrating application of our results. Enceladus model data are courtesy D. Hemingway; compare Figure S4a to Figure 11d of Hemingway and Mittal (2019). The difference in induced field magnitude relative to spherically symmetric is always positive because the largest change to the induced magnetic moments is in the dipole moment. More conducting ocean material is closer to the surface in the asymmetric model, enhancing the largest moments. Magnetic fields are evaluated at the J2000 epoch, this time at $r = 2R_M$, a plausible flyby altitude for a future mission to the Uranus system. A Seawater composition is assumed for the ocean, and a 100 km uniform ionosphere is assumed, with a total ionospheric conductance of 800 S based on comparison with the plasma environment of Callisto (Hartkorn and Saur, 2017). Electrical conductivities in the ocean are calculated using the *PlanetProfile* geophysical modeling framework (Vance et al., 2021). For simplicity, only the synodic period is modeled here. Animations for the difference in x component and magnitude as they vary throughout the synodic period are included as Supplemental Material.

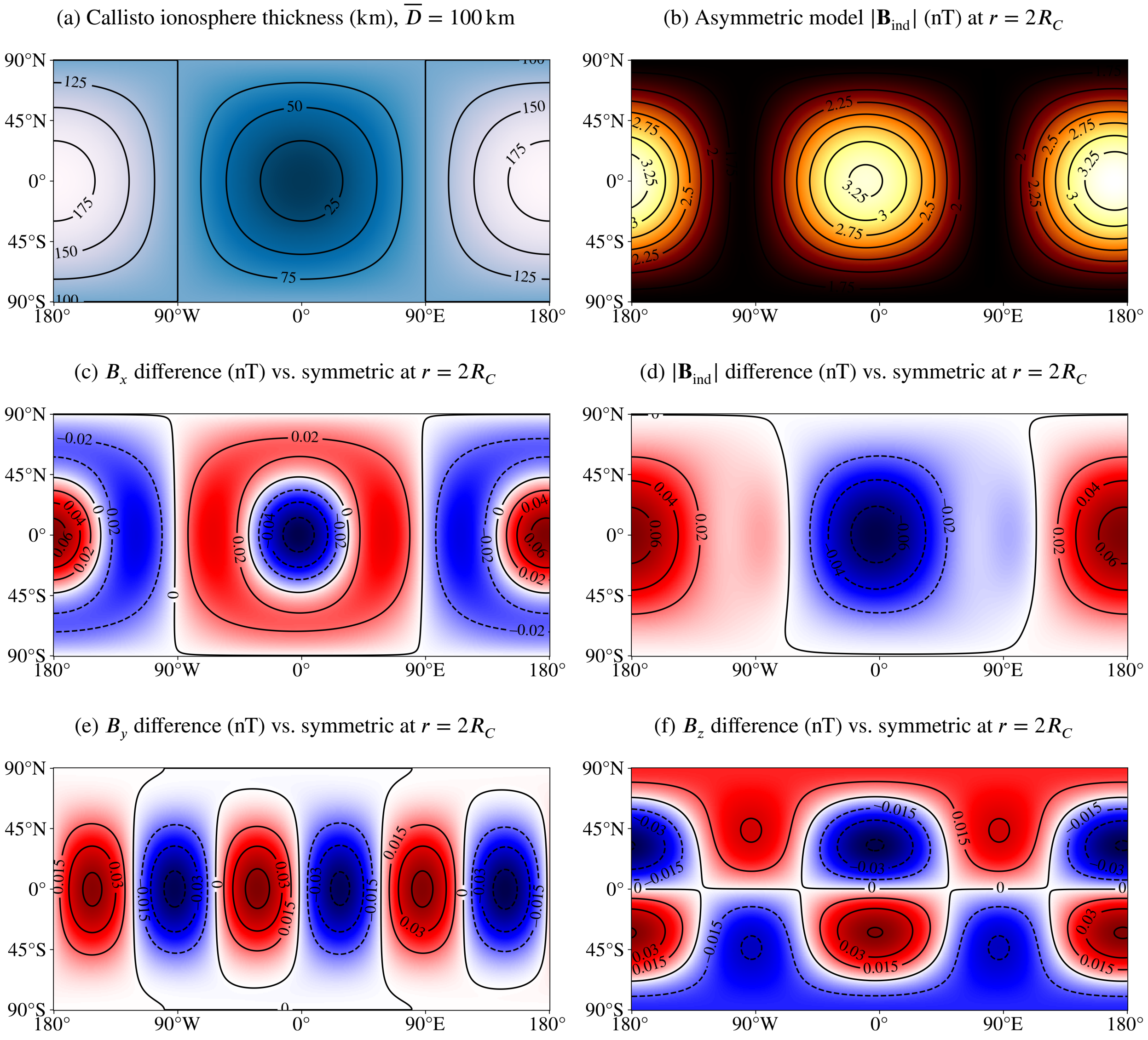

Figure S5: Callisto model with an asymmetric ionosphere modeled after Hartkorn et al. (2017). Uniform conductivity, an average ionospheric thickness of 100 km, and a total conductance of 800 S are assumed, as a rough approximation of the day–night dichotomy inferred by Hartkorn et al. An ocean with dissolved $MgSO_4$ and a 100 km thick ice shell are assumed. The magnetic field is evaluated at the J2000 epoch and at $r = 2R_C$, a plausible distance for a spacecraft flyby. In this case, the differences due to asymmetry are negligible, owing to the relatively low ionospheric conductivity where the considered asymmetry is present. Electrical conductivities in the ocean are calculated using the *PlanetProfile* geophysical modeling framework (Vance et al., 2021). For simplicity, only the synodic period is modeled here. Animations for the difference in x component and magnitude as they vary throughout the synodic period are included as Supplemental Material.

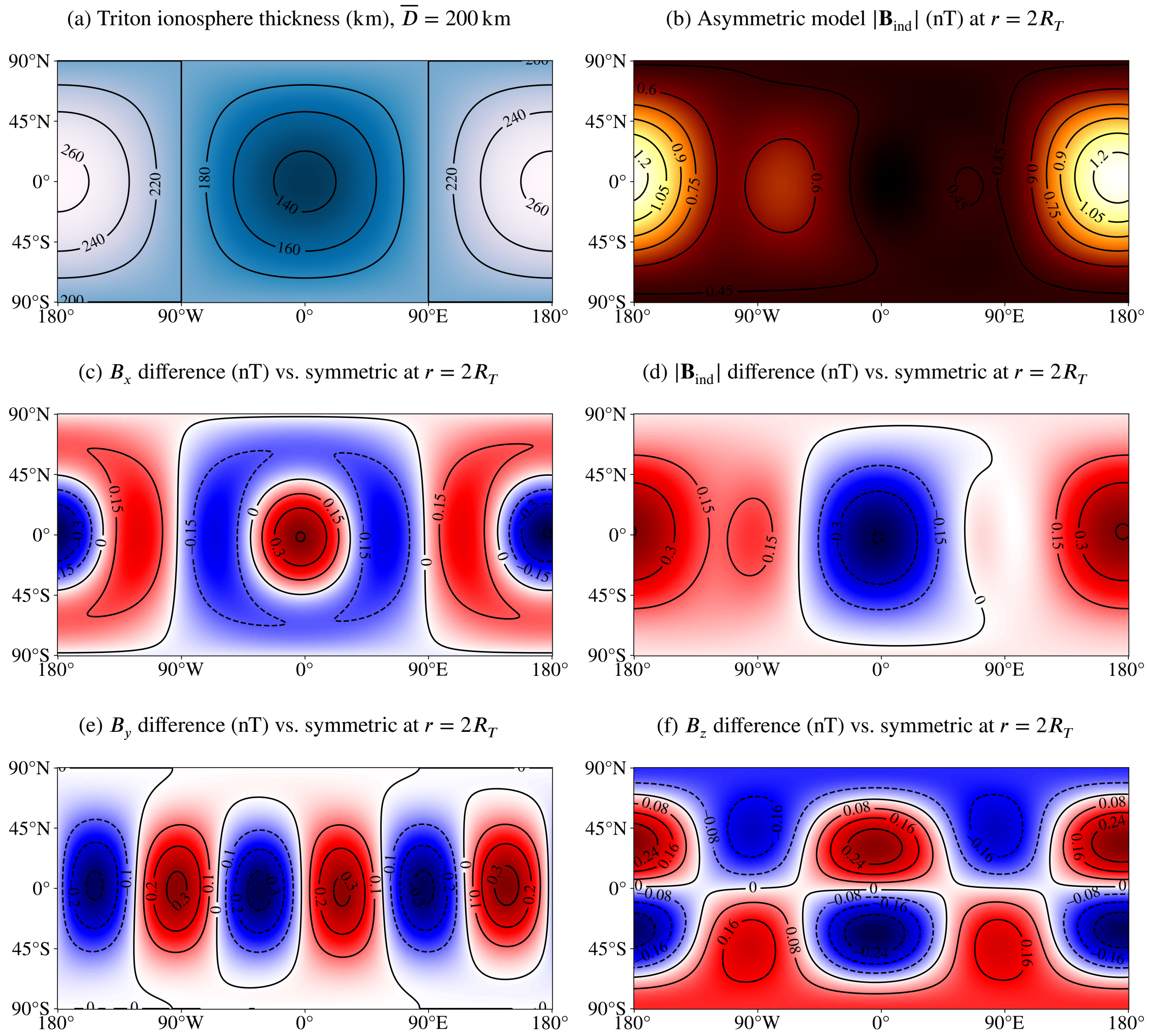

Figure S6: Triton model with an asymmetric ionosphere similar to that supposed for Callisto, an approximation of a day–night dichotomy. Uniform conductivity, an average ionospheric thickness of 200 km, a lower bound for the ionosphere of 250 km altitude, and a total height-integrated conductivity of 20 kS are assumed, based on the structure inferred by Tyler et al. (1989) from *Voyager* measurements. The interior structure we suppose for Triton is based on a moment of inertia supposed for Pluto by Hussmann et al. (2006) and geophysical modeling using the *PlanetProfile* framework. An ocean with dissolved $MgSO_4$ and a 112 km thick ice shell are assumed. The magnetic field is evaluated at the J2000 epoch and at $r = 2R_T$, a plausible distance for a spacecraft flyby. Unlike for Callisto, the differences due to asymmetry are a substantial fraction of the overall magnitude of the induced field (compare Figures S6c–S6f to S6b), owing to the high conductivity and pronounced asymmetry we suppose for the ionosphere. Electrical conductivities in the ocean are determined using the *PlanetProfile* geophysical modeling framework (Vance et al., 2021). For simplicity, only the synodic period is modeled here. Animations for the difference in x component and magnitude as they vary throughout the synodic period are included as Supplemental Material.

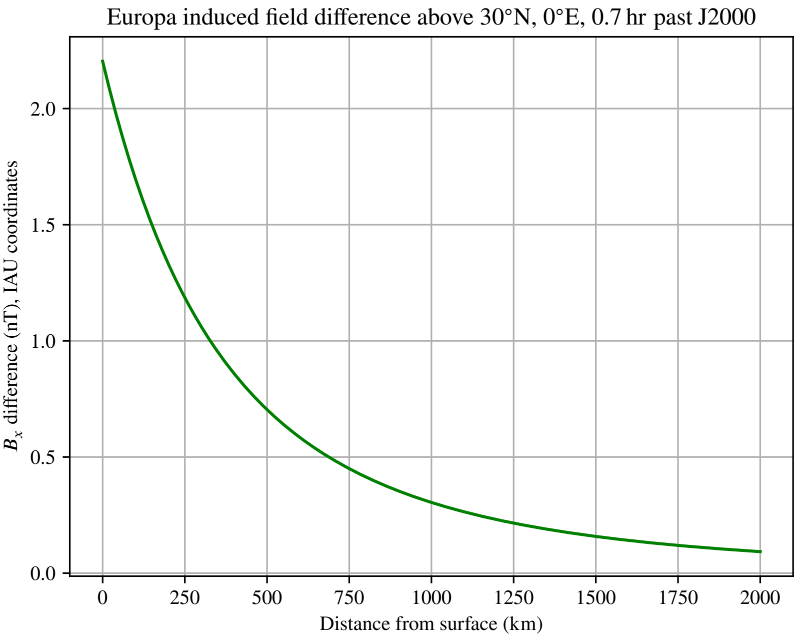

Figure S7: Difference in induced field B_x component for our Europa Seawater model as a function of altitude for a fixed point in time. The selected surface location (30°N, 0°E) and time (0.7 hr past J2000) maximize the observed difference relative to the spherically symmetric case for this interior model and component—see Figure 2. Beyond about 1500 km altitude, the difference is around 0.2 nT and likely negligible. Repeated from the main text (Figure 3).

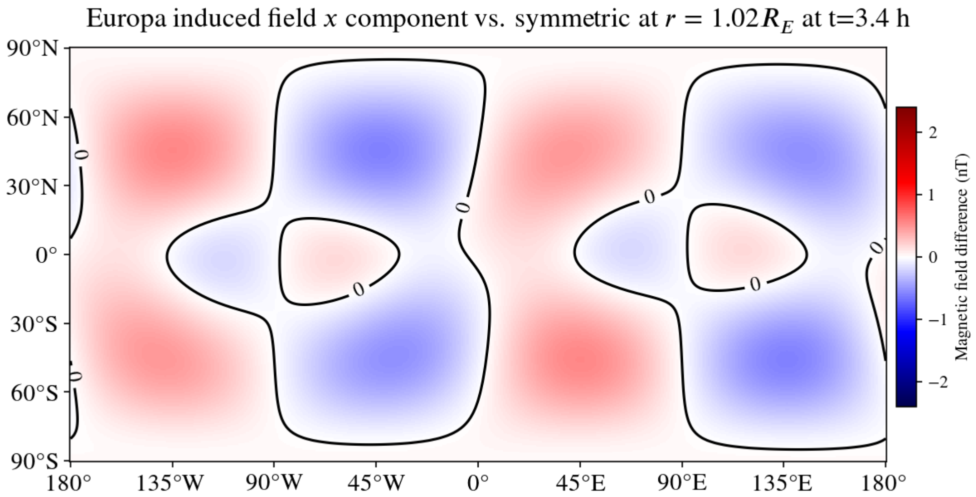

Figure S8: Single frame from the animated version of Figure S1c, at $t = 3.4$ hr past J2000. At this time, the difference resulting from asymmetry is much smaller than at J2000, and is less than 0.6 nT everywhere. This state lasts for around 30 min. A spacecraft reaching its closest approach altitude above 25 km during a flyby at times such as this may be able to ignore the influence of asymmetry under certain conditions.
